# Combinatorial Tuning of Multigene Biosynthetic Pathways based on RNA Interference

**DOI:** 10.1002/advs.78608

**Published:** 2026-11-03

**Authors:** Zixu Zhang, Yingshuang Xu, Zijia Li, Fei Du, Wang Ma, Guang Yang, Xiaoman Sun

**Affiliations:** ^1^ State Key Laboratory of Microbial Technology Nanjing Normal University Nanjing People's Republic of China; ^2^ School of Food Science and Pharmaceutical Engineering Nanjing Normal University Nanjing People's Republic of China

## Abstract

Fine‐tuning the expression level of multigene pathways remains a major challenge in engineering eukaryotic microbial cell factories. Modifications to individual pathway genes and CRISPR‐based approaches often require repeated genome reconstruction, large regulatory components, or complex guide‐array assembly. Here, we developed a lightweight, post‐transcriptional RNAi‐based strategy that enables fine‐tuning gene expression without iterative genome editing. Size‐selected small‐RNA sequencing and functional testing were used to identify endogenous Pol III promoters spanning graded transcriptional strengths in *Schizochytrium* sp. Combining promoter strength with multiple short hairpin RNA (shRNA) target positions expanded the accessible repression range and enabled high‐resolution gene‐dosage tuning. The RNAi‐based combined regulation strategy was applied to the three‐gene carotenoid pathway of *Schizochytrium* sp. and the nine‐gene docosahexaenoic acid (DHA) pathway of *Yarrowia lipolytica*, significantly enhancing the product production in both hosts. Integration of RNAi screening and dosage optimization further identified and tuned bypass pathways, increasing the ratio of DHA to docosapentaenoic acid (DPA) by 4.50‐fold versus wild type. These gains were maintained during 5‐L fermentation and serial passaging. This work establishes a compact and scalable regulatory framework for engineering complex biosynthetic pathways in eukaryotic microbial hosts.

## Graphical abstract

Small RNA sequencing identifies candidate endogenous Pol III promoters, and subsequent fluorescence screening measures knockdown efficiency to assess promoter strength. Combining promoter strength and shRNA target position enables fine‐grained RNAi regulation of multigene pathways. This strategy enhances β‐carotene production in *Schizochytrium* sp. and DHA production in *Yarrowia lipolytica*, while bypass pathway screening and tuning improve the DHA/DPA ratio in *Schizochytrium* sp.

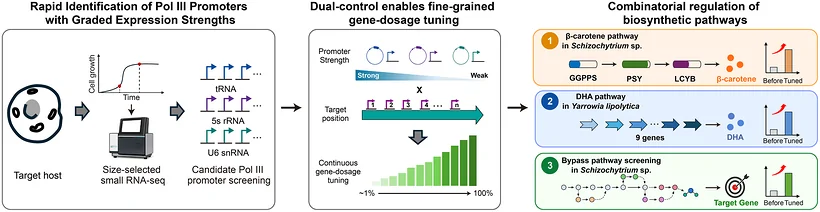

## 1 Introduction

Reprogramming metabolic pathways is the key to constructing excellent cell factories [1]. To achieve the optimal yield of the target product, it is necessary to reasonably regulate the expression of pathway genes to balance flux, alleviate metabolic bottlenecks, and minimize cellular burden [2, 3]. There are abundant expression elements and operational techniques for optimizing the expression level of a single gene [4, 5, 6], but optimizing the expression level of a full metabolic pathway remains a challenge. Because it involves the combined regulation of multiple genes, it is impossible to determine at what level each key gene should be expressed in order to achieve the goal.

Currently, one of the mainstream methods for combined regulation of the entire metabolic pathway focus on genome rewriting at the DNA level. For instance, optimizing the copy number combination of genes in a pathway [7]; shuffling upstream promoter elements and terminators through Cre‐LoxPsym recombination for in vivo expression diversification [8]; using CRISPR‐guided base editing to generate gene combinations with different ribosome binding sites, 5' untranslated regions, or promoters [9]; integrating gene expression cassettes into chromosomal loci with varying transcriptional strength [10]. Although these strategies provide a basis for pathway dosage control, they are fundamentally limited by the necessity for multiple times of genome manipulation, rendering the process labor‐intensive and time‐consuming. Furthermore, as the number of regulated genes increases, the construction quantity, editing efficiency, available sites, and genome stability assessment will make the combined regulation a highly demanding process, making it difficult to apply to hosts that do not have efficient gene editing tools. Therefore, there is a clear need for a more lightweight regulatory layer, which enables synchronous tuning of multiple gene expression levels without repeatedly constructed expression cassettes and genome manipulation.

Transcriptional and post‐transcriptional regulatory approaches offer alternative ways to reduce this construction burden. They mainly include three basic tools: CRISPRi/a technology (at the transcriptional level) [11], antisense RNA (at the post‐transcriptional level, primarily prokaryotic) [12], and RNAi (at the post‐transcriptional level, primarily eukaryotic) [13]. They have been widely applied in screening effective targets within metabolic networks, or have been used for independently regulating the expression levels of target genes [14, 15, 16, 17]. For example, in hosts such as *Escherichia coli* and *Corynebacterium glutamicum*, antisense RNA has been widely deployed to identify key targets governing product synthesis and to fine‐tune metabolic flux, leading to markedly enhanced titers of cadaverine, indigoidine, valerolactam, and so on [18, 19]. CRISPRa has been successfully applied to the combined regulation of eight genes in *Saccharomyces cerevisiae*, resulting in a 37‐fold increase in the production of squalene and a 17‐fold increase in the production of heme [20]. Nevertheless, CRISPRi/a systems require delivery of Cas effectors protein, nuclear localization, and consideration of PAM constraints, and often involve complex sgRNA array assembly [11, 21]. RNAi uses compact short hairpin RNAs (shRNAs) comprising a 21–23‐nt targeting sequence, a 6–9‐nt loop, and the reverse complement of the targeting sequence. Many eukaryotes retain the Dicer and RNA‐induced silencing complex (RISC) machinery [22]. RNAi has been successfully applied in diverse eukaryotic microbes for gene knockdown, target identification, and dynamic control [23, 24], as well as for combinatorial regulation of multiple genes to improve carotenoid production in *S. cerevisiae* [25]. In principle, RNAi‐mediated regulation offers a lightweight and scalable approach to fine‐tuning pathway gene dosage, reducing the need for repeated reconstruction of pathway‐gene expression cassettes and avoiding the delivery and assembly demands associated with CRISPR/Cas components. However, many eukaryotic microbes lack well‐characterized small‐RNA expression elements, and achieving finely graded repression of individual genes remains challenging for combinatorial pathway regulation.

In this study, we developed versatile RNAi‐based platform for combinatorial post‐transcriptional regulation of multigene pathways. To address the lack of systematic characterization of Pol III promoters in eukaryotic microbes, we used size‐selected small‐RNA sequencing and functional testing to identify endogenous Pol III promoters spanning graded transcriptional strengths. Using 13 genes from *Schizochytrium* sp. as targets, we systematically dissected the contributions of promoter strength and shRNA target‐position selection to RNAi knockdown efficiency and associated phenotypes. Building on these insights, we established a promoter–target‐position dual‐control strategy that expands the accessible repression range and enables fine‐grained expression tuning. The platform was applied to a three‐gene carotenoid pathway in *Schizochytrium* sp. and to the optimization of a nine‐gene docosahexaenoic acid pathway in *Yarrowia lipolytica*. Furthermore, we integrated RNAi target screening with dosage optimization to identify and tune bypass pathways that increase the DHA/DPA ratio while restoring lipid titer, and evaluated performance during 5‐L fermentation and serial passaging. Unlike CRISPRi/a, which requires Cas protein expression and PAM‐compatible target sites and often relies on complex sgRNA arrays, this RNAi‐based platform enables fine‐grained regulation of individual genes by combining promoter strength with shRNA target‐position selection, while supporting concurrent regulation of multiple genes. It therefore provides a lightweight route to scalable pathway‐level dosage tuning that is particularly useful for genetically less tractable eukaryotic microbial hosts.

## 2 Results

### 2.1 Identification of Pol III Promoters with Different Expression Strength

The efficient expression of shRNAs is central to harnessing RNAi in eukaryotes. In many eukaryotic microbes where research is still insufficient, there is a lack of systematically identified and functionally characterized Pol III promoters. The traditional methods mostly rely on direct prediction based on genomic annotations, which have problems such as low accuracy, lack of experimental verification, and uncontrollable expression intensity [26]. Here, we developed a streamlined pipeline for the discovery and functional characterization of Pol III promoters based on size‐selected small RNA sequencing (Figure 1A). First, small RNA sequencing was conducted on samples at different time points. Within each of three representative classes of Pol III‐transcribed small RNAs, candidate promoters were selected based on the transcriptional levels of their corresponding small RNAs and whether these levels remained relatively stable between the two time points. Subsequently, each candidate promoter was used to drive an *eGFP*‐targeting shRNA, and the resulting construct was introduced into the target host. Finally, the knockdown efficiency of the shRNAs driven by each promoter was quantitatively evaluated using the fluorescence intensity of the reporter gene, thereby ranking the promoter strengths. Applying this workflow to *Schizochytrium* sp., we isolated candidates whose transcriptional output remained within a 1.5‐fold variation between 24 and 72 h. These included three distinct classes, tRNA, 5S rRNA, and U6 snRNA, with tRNA‐derived promoters representing the most abundant category (Figure 1B and Figure S1). As shown in Figure 1C, the candidate promoters at different transcriptional levels exhibit a significant gradient in knockdown efficiency (Figure S2). The high‐expression promoter of tRNA‐Gln (TTG)‐1 has an inhibition efficiency of over 95%, while the weak‐expression promoter of 5S rRNA‐20 is only about 8.46%. However, the commonly used hU6 promoter failed to function in *Schizochytrium* sp. We observed that knockdown efficiency for all six tested promoters declined at 60 and 96 h compared to 24 h (Figure 1D). This might be related to the fact that the *Schizochytrium* sp. lacks a natural RNA‐dependent RNA polymerase (RdRP), which is responsible for the amplification and signal amplification of dsRNA precursors in the RNAi pathway. Supporting this, the introduction of an exogenous RdRP from *Schizosaccharomyces pombe* rescued the phenotype, increasing knockdown efficiencies at 96 h by approximately 20% across all tested promoters, significantly improving the inhibition stability over the time period from 24 to 96 h (Figure 1D). In summary, by combining size‐selected small RNA sequencing with functional verification, a series of Pol III promoters with different expression intensities can be obtained.

**FIGURE 1:**
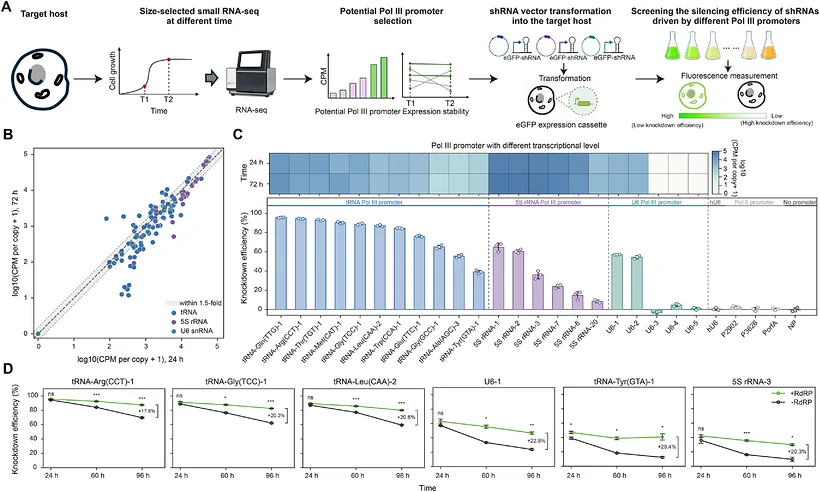
Identifying and validating graded‐strength Pol III promoters. (A) Scheme of the size‐selected small RNA sequencing used to obtain Pol III promoters. (B) the log10(CPM per copy + 1) was analyzed at 24 h vs 72 h. Points within 1.5‐fold (gray band) indicate stable expression. (C) Knockdown efficiency (%) of shRNAs driven by Pol III promoters with different transcriptional level, hu6, and Pol II promoters. (D) Knockdown efficiency (%) at three time points for 6 promoters with (+RdRP) or without (−RdRP). Statistical comparisons were performed between the +RdRP and −RdRP groups carrying the same promoter at each time point. ns, not significant; *p < 0.05; **p < 0.01; ***P < 0.001. In C‐D, data are presented as the mean ± s.d. of three independent transformants per construct.

### 2.2 Comparison of Target Position Selection and Promoter Strength for Fine‐Grained Gene Expression Control

Achieving fine‐grained control over target gene expression is pivotal for precise metabolic engineering [27]. Adjusting the knockdown efficiency of RNAi by selecting different target sequence positions is a common approach, but this method has limited regulatory scope and precision [28]. We systematically compared the effects of Pol III promoters with different expression strength and different target positions on the efficiency of target gene silencing and the phenotype. We selected 13 endogenous genes involved in lipid biosynthesis in *Schizochytrium* sp. (Figure 2A). For each gene, we compared three target positions under a fixed promoter and three promoter strengths at target position 1. Results demonstrate that target positions selection significantly influences knockdown efficiency; however, this effect varies substantially across genes. For instance, the knockdown range for *CPT1* gene spanned from 38.3% to 84.6%, whereas for gene *DGL*, it was restricted to a narrow window between 80.9% and 87.9% (Figure 2B). It is worth noting that even though there are small differences in inhibition efficiency, significant phenotypic differences have emerged. For instance, the knockdown efficiency of *ORFC* gene at positions 1 and positions 3 was 89.5% and 85.1% respectively (the difference is very small), but the differences in biomass and lipid titers caused by this are from 71.0 g/L and 15.1 g/L to 89.2 g/L and 20.5 g/L, respectively (Figure 2C). Similarly, the *ACOX1* and *TGL* gene also exhibited the similar phenomenon. This further demonstrates the necessity of fine‐grained regulation of gene expression.

**FIGURE 2:**
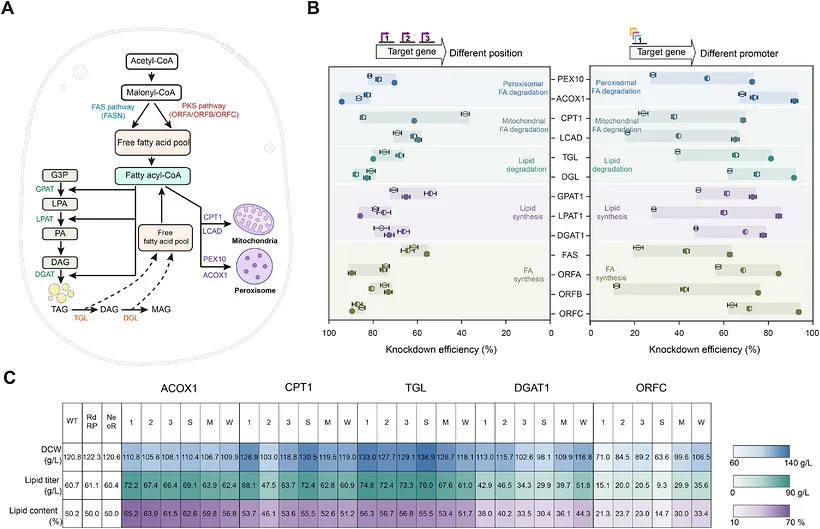
Comparison of the effect of target position selection and promoter strength on knockdown efficiency and phenotype. (A) 13 genes related to lipid synthesis. (B) Knockdown efficiency (%) of the 13 targets across different target positions and promoters. In the target‐position panel, target sites 1, 2 and 3 are shown as solid, half‐open and open circles, respectively. In the promoter panel, S, M and W indicate strong, medium and weak promoters and are shown as solid, half‐open and open circles, respectively. (C) DCW, lipid titer, and lipid content produced after inhibiting 5 genes at different positions (1, 2, and 3) and using different promoters (S, strong; M, medium, and W, weak). In B‐C, data are presented as the mean ± s.d. of three independent transformants per construct.

In contrast, employing Pol III promoters of varying strengths expanded the regulatory range for 12 genes beyond that achieved by positional tuning alone (Figure 2B). Specifically, for *ORFB* and *ORFC*, the knockdown ranges were extended from 73.1%–80.7% and 85.1%–89.5% (position groups) to 11.9%–75.4% and 63.8%–93.6% (promoter groups), respectively. This demonstrates that promoter strength gradients offer a broader dynamic range than target positions selection. However, neither relying on position control or promoter strength alone can achieve continuous, fine‐grained control spanning the full spectrum from near‐zero to near‐complete silencing (∼1%–100%).

### 2.3 Dual Control of the Promoter and the Target Position Enables Fine‐Grained Regulation

We next investigated whether the granularity of regulation could be improved when the targeted position was fully covered. The results showed that the expression level of the reporter gene (*eGFP*) still exhibited a discontinuous distribution, and it was impossible to achieve continuous and fine coverage of the expression interval (Figure 3A). To overcome this limitation, we implemented a dual control strategy incorporating nine promoters of varying strengths and six distinct target positions (Figure 3B and Figure S3). The results showed that this promoter‐position dual control strategy enabled *eGFP* to exhibit a highly fine‐grained expression gradient, significantly superior to single‐factor regulation (Figure 3C).

**FIGURE 3:**
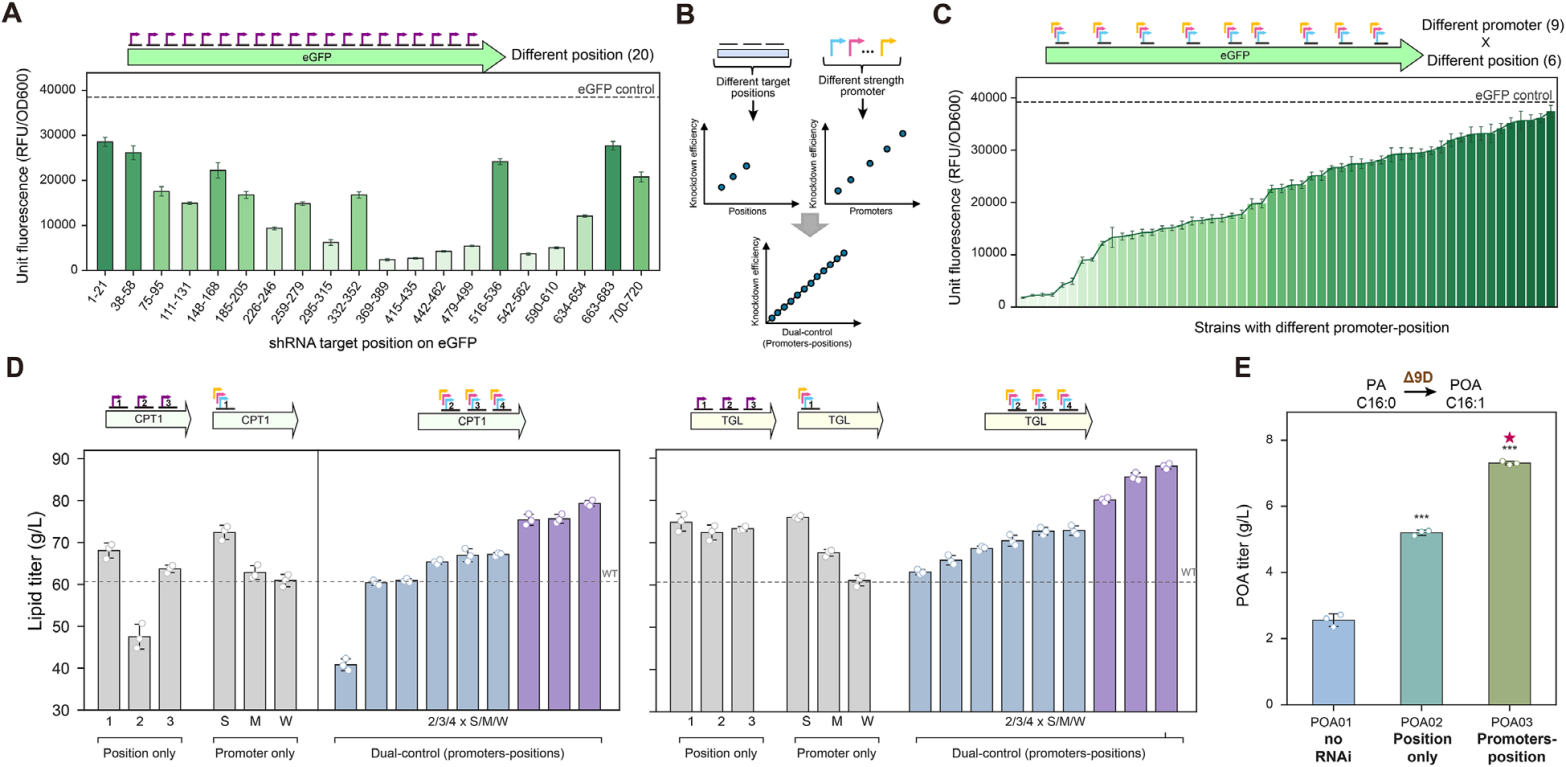
Optimization of shRNA design by dual control of target position and promoter strength. (A) unit eGFP fluorescence (RFU/OD_600_) under different target positions. Dashed line indicates the mean unit fluorescence of the without knockdown eGFP (eGFP control). (B) Conceptual diagram illustrating the dual‐control of promoter‐position strategy. (C) unit eGFP fluorescence (RFU/OD_600_) under the dual‐control of promoter strength and target position. Dashed line indicates the mean unit fluorescence of the without knockdown eGFP (eGFP control). (D) Impact of single‐control (position only or promoter only) and dual‐control (promoter‐position combinations) on lipid titer (g/L) for two target genes, *CPT1*and *TGL*. Purple bars indicate titers higher than the single‐control. Dashed lines indicate the lipid titer of the WT. (E) POA titer of POA01 (no RNAi), POA02 (position only), and POA03 (promoter‐position). Statistical comparisons were performed between POA02 and POA01 and between POA03 and POA01, with POA01 serving as the control. Significance was defined as follows: ns, not significant; *p < 0.05; **p < 0.01; ***p < 0.001. In A and C‐E, data are presented as the mean ± s.d. of three independent transformants per construct.

We then applied this strategy to regulate two genes (*CPT1* and *TGL*) again, aiming to determine whether fine‐tuning could further enhance lipid titers compared single‐control. As shown in Figure 3D, in *CPT1* and *TGL* groups, there are all three groups whose lipid titer is higher than that of single‐control group. In *TGL* group as an example, the maximum lipid titer of the dual‐control is 88.1 g L^−1^, representing an approximately 15.9% increase over the highest titer achieved with single‐control strategy (promoter‐only regulation using the strong promoter; 76.0 g/L). To verify whether this strategy can enhance the synthesis ability of specific functional fatty acids, we introduced an exogenous *Des9* (*9D*) gene from *Scheffersomyces segobiensis* into *Schizochytrium* sp., and constructed an engineered strain POA01 capable of synthesizing the functional fatty acid palmitoleic acid (POA). We subsequently introduced the shRNA combinations that yielded the highest lipid titers in the single‐control and dual‐control *TGL* groups into POA01 to construct POA02 and POA03, respectively. After fine‐grained regulation of *TGL* in this strain, the titer of POA increased to 7.31 g L^−1^, which was 1.41‐fold higher than that achieved solely through positional regulation (POA02) and 2.86‐fold higher than that without regulation (POA01), demonstrating the substantial potential of this fine‐tuning regulation strategy for metabolic engineering (Figure 3E).

### 2.4 RNAi‐Mediated Combinatorial Regulation of Three‐Gene and Nine‐Gene Pathways in Two Hosts

Achieving high‐yield accumulation while balancing the metabolic flow via the combinatorial tuning of individual gene expression levels represents a cornerstone strategy in metabolic engineering [29]. Here, we applied RNAi to combinatorial regulation of multi‐gene pathways (Figure 4A). First, target genes in a certain biosynthesis pathway are selected. Then, an RNAi library is constructed by using different strength of Pol III promoters and multiple target positions, yielding a strain library. Finally, the optimal gene expression dosage combination is obtained through phenotypic screening. If it is necessary to further increase the product titer, the metabolic pathway can be multi‐copy integrated after obtaining the optimal gene expression dosage combination.

**FIGURE 4:**
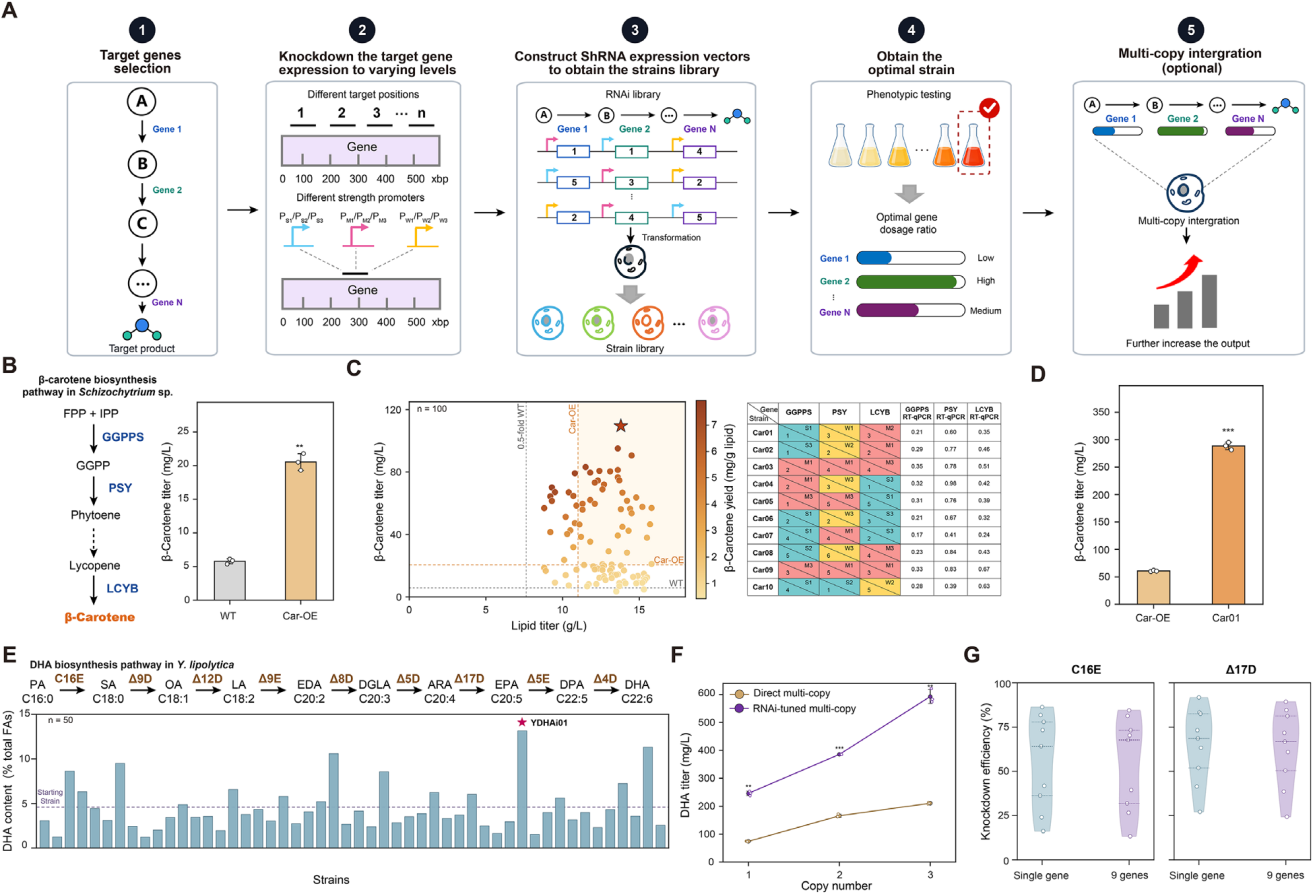
RNAi‐mediated combinatorial regulation of multi‐gene pathways in two hosts. (A) the overall workflow of RNAi‐mediated combinatorial regulation strategy for enhancing target product titers. (B) Description of the three key genes in β‐Carotene biosynthesis pathway in *Schizochytrium* sp. and the titer β‐Carotene of WT and CarOE (increasing the three key genes to 2 copies). (C) β‐Carotene titer of 100 strains (Five‐pointed star representing the top‐performing strain), and analyzing the target position, Pol III promoter, and relative expression levels of *GGPPS*, *PSY*, and *LCYB* for the top 10 strains (Car01‐10). Dashed lines indicate the strains of WT and CarOE for lipid titer and β‐carotene titer. (D) β‐Carotene titer of CarOE and Car01 in fed‐batch fermentation. (E) Description of the nine key genes in DHA biosynthesis pathway in *Y. lipolytica*. and the DHA content of 50 randomly selected strains. (F) DHA titer of multi‐copy integration using unregulated and RNAi‐mediated regulated pathway. (G) Knockdown efficiency (%) of *C16E* and *Δ17D* genes under the conditions of single‐gene and nine‐gene targets. In B and D‐G, data are presented as the mean ± s.d. of three independent transformants per construct. Statistical comparisons were performed between CarOE and WT in B, between Car01 and CarOE in D, and between the RNAi‐tuned and direct multi‐copy integration groups at the same copy number in F. Significance was defined as follows: ns, not significant; *p < 0.05; **p < 0.01; ***p < 0.001.

We applied this strategy to optimize the carotenoid biosynthesis pathway in *Schizochytrium* sp., focusing on three key genes (*GGPPS*, *PSY*, and *LCYB*). To facilitate subsequent color‐based phenotypic screening, these three genes were integrated in double copies (engineered strain CarOE), resulting in an increase in carotenoid titer from 5.79 mg L^−1^ (WT) to 20.5 mg L^−1^ (Figure 4B). A combined RNAi library was constructed, which included nine of Pol III promoters with different strength (three high, three medium, and three low each) and six target positions for each gene. Screening of 100 transformants identified a high‐performing engineered strain, Car01, which produced 18.0‐fold more carotenoids than the WT and 5.02‐fold more than the parental strain of CarOE, demonstrating the power of combinatorial tuning in balancing metabolic flux (Figure 4C). Further reverse transcription quantitative PCR (RT‐qPCR) analysis of the top ten producers showed that *GGPPS* expression was consistently reduced to 17–35% of its level in the starting strain CarOE (Figure 4C). In most strains, *PSY*, which channels GGPP into carotenoid biosynthesis, showed milder repression, while *LCYB* showed intermediate repression (Figure 4C). These diverse combinations of gene repression were associated with increased β‐carotene titers, highlighting the value of combinatorial regulation in identifying productive expression configurations across the pathway (Figure 4C and Figure S4). Car01 was further evaluated in fed‐batch fermentation, achieving a β‐carotene titer 4.75‐fold that of CarOE (Figure 4D).

To further validate the versatility of this combinational regulation strategy across different hosts and longer pathways, we tested its effect on a nine‐gene DHA synthesis pathway in *Y. lipolytica*. We constructed a RNAi library using six promoters paired with nine target positions per gene. Screening of fifty randomly selected transformants revealed that fifteen engineered strains outperformed the parental control; among them, the top producer, YDHAi01, achieved a DHA content of 13.2%, representing 2.88‐fold of the starting strain (Figure 4E). We next assessed the impact of multi‐copy pathway integration. In the unregulated group, DHA titers increased from approximately 72 mg L^−1^ at one copy to 165 and 210 mg L^−1^ at two and three copies, respectively (Figure 4f). In the combinatorial regulated group, increasing pathway copy number led to greater gains in DHA titer, with significantly higher titers than the unregulated group at each copy number (Figure 4f). At three copies, the DHA titer reached 593 mg L^−1^, 2.81‐fold that of the corresponding unregulated group. These results highlight the importance of coordinated pathway gene regulation and suggest that balancing metabolic flux is critical for realizing the full potential of multi‐copy strategies. Finally, to rule out potential crosstalk or dilution effects inherent in complex multi‐gene circuits, we examined whether the knockdown efficiency of individual genes is compromised within the nine‐gene context. We compared the knockdown efficiency of two genes, *C16E* and *17D*, under both single‐gene and nine‐gene combinatorial backgrounds. It is worth noting that there is no significant difference in the knockdown efficiency between the two scenarios. For instance, the inhibition efficiency of *C16E* under single‐gene regulation is 24.0%–81.8%, while under nine‐gene regulation it is 13.3%–84.4% (Figure 4G). These results confirm that our RNAi‐based combinatorial strategy maintains high stability, even when scaling up to long biosynthetic pathways.

### 2.5 Integration of Screening and Fine‐Tuning of Key Genes in the Bypass Pathways

RNAi has been widely applied in the screening of target genes [24]. Here, we extend its utility to the combinatorial regulation of bypass pathways (Figure 5A). This method first identifies the alternative pathways that may affect the synthesis of the target product. Then, it independently inhibits each candidate gene using RNAi, and selects the key genes that have a significant impact on the target product. On this basis, it finely regulates the RNAi of these key genes to obtain the optimal gene expression dosage.

**FIGURE 5:**
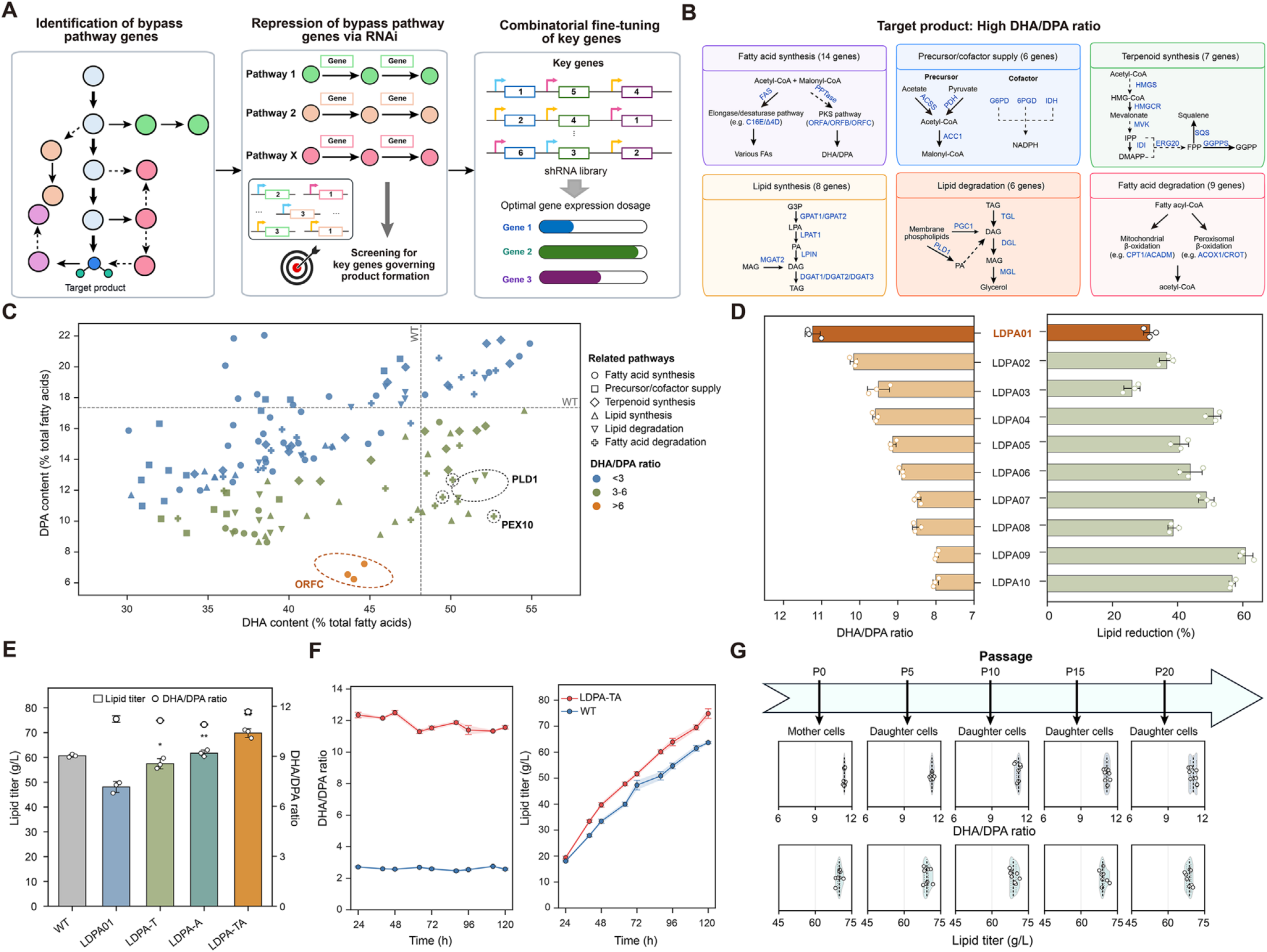
Integration of the screening and fine regulation in the bypass pathways. (A) the overall workflow of RNAi‐mediated screening and fine‐tuning key genes for increasing target product titers. (B) the bypass pathways related to high DHA/DPA ratio products and their 50 candidate targets. (C) DHA and DPA contents following individual knockdown of 50 bypass genes, identifying three key regulatory genes (*ORFC*, *PEX10*, and *PLD1*). Dashed lines indicate the corresponding WT strain (the control group) of DHA and DPA contents. (D) DHA/DPA ratio and lipid reduction of top 10 strains. (E) Lipid titer and DHA/DPA ratio following precise regulation for *TGL* and *ACOX1*. Strains that single‐gene knockdown (LDPA‐T for *TGL*, LDPA‐A for *ACOX1*) and double‐gene knockdown (LDPA‐TA) were evaluated. All statistical comparisons were performed using LDPA01 as the control. (F) DHA/DPA ratio and lipid titer of WT and LDPA‐TA strains. (G) The knockdown stability testing procedure and results by serial passaging. In D‐F, data are presented as the mean ± s.d. of three independent transformants per construct. Significance was defined as follows: ns, not significant; *p < 0.05; **p < 0.01; ***p < 0.001.

Ultra‐high purity DHA is critical for pharmaceutical applications; however, the co‐presence of DPA significantly compromises downstream separation efficiency and increases production costs. Therefore, engineering microbial strains to produce lipid with a high DHA/DPA ratio is of paramount importance. In *Schizochytrium* sp., both DHA and DPA are synthesized via the polyketide synthase (PKS) pathway, yet the mechanism governing carbon flux allocation between them remains elusive [30].

To address this, we systematically analyzed six related bypass pathways related to DHA and DPA synthesis, screening 50 candidate genes (Figure 5B). Results showed that individual knockdown of *ORFC*, *PLD1* and *PEX10* can increase the DHA/DPA ratio (Figure 5C and Figure S5). We subsequently applied nine promoter strengths and six target positions per gene to fine‐tune three genes (Figure S6). Among the top 10 engineered strains selected from 100 transformants, LDPA01 achieved the highest DHA/DPA ratio of 11.2, which was 4.06‐fold that of the WT (Figure 5D). However, this optimization came at the cost of reduced total lipid titer (Figure S7). To resolve this trade‐off, we carried out precise single‐gene (LDPA‐T and LDPA‐A) and double‐gene (LDPA‐TA) regulation for *TGL* and *ACOX1* respectively in LDPA01. Results showed that the lipid titer of the double‐gene regulation was higher than that of the single‐gene regulation, and successfully restored to WT level without compromising the DHA/DPA ratio (Figure 5E). In a 5 L bioreactor, LDPA‐TA achieved a DHA/DPA ratio of 11.6, representing a 4.50‐fold increase over the WT (Figure 5F). Finally, we assessed the genetic stability of the RNAi‐mediated regulation over multiple passage numbers. Compared to the initial passage number (P0), the P20 daughter cells showed no significant decline in DHA/DPA ratio, lipid titer, or target gene silencing efficiency (Figure 5G and Figure S8). This confirms that our combinatorial RNAi strategy offers robust genetic stability.

## 3 Discussion

Constructing an excellent cell factory often involves multiple enzymes working together. Fine‐tuning the expression of individual pathway genes has been proven crucial for optimizing production and limiting by‐product formation [2, 3]. However, rational tuning of expression levels of a full metabolic pathway has proven challenging. It usually relies on repeatedly modifying individual pathway genes (such as promoter replacement, copy number control, etc.) or through transcriptional control mediated by CRISPRi/a [31, 32]. Nevertheless, these strategies rely on the host's regulatory element library and editing efficiency, thereby confining most applications to well‐characterized model hosts such as *E. coli* and *S. cerevisiae* [8, 10, 20, 33, 34, 35]. Here, we have developed a combinatorial regulation method based on RNAi, which enables fine‐grained repression of pathway genes without the need for repeatedly reconstructing of chromosomal expression cassettes, rendering it particularly advantageous for genetically intractable eukaryotic microbial hosts.

The RNAi mechanism involves the processing of shRNAs by Dicer into small interfering RNAs (siRNAs), followed by the loading of the guide strand into an Argonaute‐containing RISC, which subsequently directs the recognition and degradation of complementary target mRNAs [13]. Consequently, the development of RNAi requires addressing two core challenges: first, whether the host possesses endogenous functional components such as Dicer and Argonaute; and second, whether shRNAs can be expressed stably and efficiently within the target host. Regarding the first challenge, many eukaryotic microbes retain RNAi‐related machinery, providing a basis for evaluating this strategy in additional hosts [36, 37]. Furthermore, even in hosts lacking certain core components, studies have demonstrated that RNAi function can be reconstituted via the heterologous introduction of elements like Dicer or Argonaute. For instance, functional RNAi has been established in *S. cerevisiae* and *Y. lipolytica* through the introduction of *Dcr1* (Dicer) and *Argo1* (Argonaute) [38, 39]. Addressing the second challenge, previous efforts to express small RNAs often relied directly on U6 or other reported small RNA promoters [23, 40]; however, these elements are not universally applicable to novel target hosts. We propose a host‐customization workflow that mines endogenous Pol III promoters through sequencing and genome annotation, followed by experimental screening to obtain a series of promoters with varying expression strengths (Figure 1A). Using *Schizochytrium* sp. as a case study, which is a typical non‐model eukaryotic microorganism, we demonstrated that the conventionally used U6 promoter is suboptimal, whereas tRNA‐derived promoters drive significantly more effective shRNA expression (Figure 1C). These findings highlight the pronounced host‐dependence of small RNA expression elements, underscoring that the identification of robust Pol III promoters is pivotal to the efficacy of combinatorial regulation strategies.

Furthermore, a critical consideration in combinatorial regulation is the number of genes that can be simultaneously targeted. Multiplex CRISPRi requires the expression of multiple sgRNAs, where large sgRNA arrays often introduce repetitive sequences, uneven guide expression, and additional assembly burdens [41]. Although strategies such as tRNA‐sgRNA and Csy4 have significantly mitigated these issues, multi‐gRNA arrays still increase construct complexity and may lead to expression heterogeneity or array instability [42, 43, 44]. In contrast, RNAi relies on a considerably more compact shRNA targeting module for each gene, only comprising a targeting sequence, a loop, and the reverse complement of the targeting sequence. Notably, even when this RNAi framework was applied to a pathway comprising nine genes, the dynamic range for regulating individual genes remained indistinguishable from that achieved when targeting a single gene alone (Figure 4G). RNAi has been exploited to tune the expression of 10 genes [25]. In that study, the shRNA expression cassettes were integrated into the δ sites of the *S. cerevisiae* genome (where multiple copies can be obtained in a single transformation), such that differing shRNA copy numbers yielded different strengths of RNAi. However, because δ sequences are yeast‐specific, this strategy is difficult to transfer to other hosts. Here, this study controls RNAi strength by coupling Pol III promoters of varying strengths with the selection of target position, without relying on any host‐specific genomic feature; it therefore holds cross‐host generalization potential.

Naturally, the application of RNAi to combinatorial regulation still faces certain limitations. First, the current Pol III‐shRNA framework is primarily constitutive and lacks the convenient inducible switches afforded by controlling Cas protein expression in CRISPRi [45, 46], thus restricting its utility in metabolic engineering scenarios requiring dynamic or stage‐specific regulation. Furthermore, the simultaneous expression of multiple shRNAs may lead to off‐target suppression, guide competition, saturation of Dicer or Argonaute, and cellular fitness burdens; these challenges become increasingly pronounced as the number of regulated genes expands. In summary, this study establishes a post‐transcriptional combinatorial regulation framework based on RNAi. Given its reliance on conserved RNAi machinery, his technique may be potentially expandable to other eukaryotic microbial hosts. It offers a simple yet practical avenue for balancing complex pathways and serves as a valuable complement to existing repetitive modifications to individual pathway genes and CRISPR‐based metabolic engineering strategies.

## 4 Methods

### 4.1 Strain and Plasmid

*Schizochytrium* sp. HX‐308 was maintained in our laboratory and was deposited at the China Center for Type Culture Collection (CCTCC) under accession number CCTCC M 209059. The starting strain *Y. lipolytica* YLPDHA02 was constructed in our previous study [47]. *Agrobacterium tumefaciens* AGL‐1 was maintained in our laboratory. *E. coli* DH5α (Tsingke Biotech, TSC‐C14) was used for plasmid construction and propagation. Strains and plasmids used in this study were listed in Table S1.

All shRNA expression plasmids were constructed by Golden Gate assembly. Each shRNA cassette contained a 21‐nt target sequence, the 9‐nt loop sequence TTCAAGAGA and the 21‐nt reverse complement of the target sequence. The 21 bp shRNA target sequences and Pol III promoters used in this study are listed in Tables S2 and S3, respectively. In *Schizochytrium* sp., the different RNAi plasmids contained no more than six assembled fragments, corresponding to three complete shRNA expression cassettes. Each cassette contained one Pol III promoter and one shRNA‐terminator fragment. Target fragments carrying *Bsa*I restriction sites (5′‐GGTCTCN‐3′), including Pol III promoters and shRNA‐terminator fragments, were digested and ligated into pZPK‐shRNA vector (maintained in our laboratory and are listed in Table S1) carrying different selectable markers using T4 DNA ligase (Vazyme Biotech, C301‐01). For *Y. lipolytica*, the RNAi plasmids contained more fragments. These plasmids were constructed using the three‐stage assembly method described in our previous study [7]. The overexpression plasmids carrying the complete DHA biosynthesis pathway were constructed using the same three‐stage assembly method.

Other overexpression plasmids used in *Schizochytrium* sp. were constructed by Gibson assembly. The gene sequences and primers are listed in Tables S4 and S5, respectively. DNA fragments were amplified using 2× Phanta UniFi Master Mix (Dye Plus) (Vazyme Biotech, P526). PCR products and restriction‐digested linearized vectors were purified using a DNA Extraction Mini Kit (Vazyme Biotech, DC301). The purified fragments were assembled using the ClonExpress Ultra One Step Cloning Kit V3 (Vazyme Biotech, C117), and the assembled plasmids were transformed into *E. coli* DH5α. Transformants were checked by colony PCR. All constructed plasmids were verified by DNA sequencing.

### 4.2 Strain Culture Condition and Transformation

For *E. coli* and *A. tumefaciens*, cells were cultured in Luria‐Bertani (LB) medium containing 10 g L^−1^ tryptone, 5 g L^−1^ yeast extract and 10 g L^−1^ NaCl. When required, LB medium was supplemented with 100 mg L^−1^ ampicillin or 50 mg L^−1^ kanamycin according to the selectable marker carried by the plasmid. *E. coli* was cultured at 37°C for 12–14 h, and *A. tumefaciens* was cultured at 28°C for 16–20 h.

For *Schizochytrium* sp., the seed and fermentation media were similar to those used in a previous study [48]. The seed medium was prepared with artificial seawater and contained 50 g L^−1^ glucose, 10 g L^−1^ monosodium glutamate and 4 g L^−1^ yeast extract. Vitamins B1, B6, and B12 were each added at 0.25 mg L^−1^, and the trace element mixture was added at 1 g L^−1^. The fermentation medium contained 100 g L^−1^ glucose, 10 g L^−1^ yeast extract and 20 g L^−1^ monosodium glutamate, while the other components were the same as those in the seed medium. *Schizochytrium* sp. cultures were grown in 250 mL flasks containing 50 mL medium at 28°C and 170 rpm. For fed‐batch culture, glucose was added at 48, 72, and 96 h to restore the glucose concentration to 80 g L^−1^. *Schizochytrium* sp.transformation was performed by *A. tumefaciens*‐mediated transformation as described in our previous study. According to the selectable marker carried by the pZPK vector, transformants were cultured for two passages in GPYS medium (50 g L^−1^ glucose, 10 g L^−1^ tryptone, 5 g L^−1^ yeast extract and 20 g L^−1^ sea salt) containing G418 (1 g L^−1^), nourseothricin (300 mg L^−1^), Zeocin (100 mg L^−1^) or hygromycin B (1 g L^−1^). Positive transformants were confirmed by colony PCR.

For *Y. lipolytica*, cells were cultured in YPD medium containing 20 g L^−1^ glucose, 20 g L^−1^ peptone and 10 g L^−1^ yeast extract. Synthetic defined (SD) agar medium containing 5 g L^−1^ (NH4)_2_SO4, 20 g L^−1^ glucose, 1.7 g L^−1^ yeast nitrogen base without amino acids and 30 g L^−1^ agar was used to select *Y. lipolytica* transformants. YPD agar medium containing 5‐FOA was used to remove the *URA3* counter‐selection marker. The fermentation medium used for DHA titer assays contained 2.5 g L^−1^ yeast extract, 0.3 g L^−1^ peptone, 1.7 g L^−1^ yeast nitrogen base without amino acids and 60 g L^−1^ glucose, and fermentation was carried out for 72 h. *Y. lipolytica* transformation was performed by chemical transformation using the Frozen‐EZ Yeast Transformation II Kit (Zymo Research, Orange). Linearized plasmids were introduced into *Y. lipolytica* after digestion with *Eco*RI and *Not*I.

### 4.3 Size‐Selected Small RNA Sequencing and Analysis

For size‐selected small RNA sequencing, *Schizochytrium* sp. samples were collected at 24 and 72 h. Total RNA samples were sent to CloudSeq Biotech Inc. (Shanghai, China) for library construction, sequencing and primary bioinformatics analysis. Small RNAs shorter than 200 nt were enriched from total RNA using the mirVana miRNA Isolation Kit (Thermo Fisher Scientific, AM1560). The enriched small RNAs were treated with AlkB enzyme mix at 37°C for 100 min. Small RNA libraries were constructed using the GenSeq Small RNA Library Prep Kit (GenSeq, Inc.) according to the manufacturer's instructions. Libraries within the small‐RNA size range were selected and sequenced on an Illumina NovaSeq platform. Raw reads were generated after sequencing, base calling and quality filtering. Sequencing quality was evaluated using Q30. Adapter sequences and low‐quality reads were removed using Cutadapt v1.9.3, and reads with a length of at least 15 nt were retained for further analysis. A small RNA reference database was built from annotated *Schizochytrium* tRNA, 5S rRNA and U6 snRNA sequences. For tRNAs, CCA was added to the 3′ end before read mapping. Trimmed reads were aligned to the small RNA reference database using Bowtie2 v2.2.4. Reads mapped to each small RNA sequence were counted using SAMtools v1.3.1. edgeR was used for count normalization and expression analysis.

### 4.4 Fluorescence Assay

For fluorescence assays, *Schizochytrium* sp. engineered strains carrying *eGFP* (308‐eGFP) alone or both *eGFP* and an *eGFP*‐targeting shRNA construct were cultured in seed medium. Samples were collected at 48 h. Fermentation broth samples were centrifuged at 1000 × *g* for 2 min, and the supernatant was removed. Cell pellets were resuspended and diluted tenfold with phosphate buffered saline (PBS) buffer (pH7.4). The diluted samples were transferred into black opaque 96‐well microplates (Corning, 3603). eGFP fluorescence and OD_600_ were measured using a Synergy H1 hybrid multi‐mode microplate reader (BioTek). eGFP fluorescence was measured with excitation and emission wavelengths of 485 nm and 520 nm, respectively. OD_600_ was measured in the same samples to normalize cell density.

### 4.5 Pol III Promoter Screening

To identify potential Pol III promoters with stable expression, CPM values were calculated for each unique small RNA sequence. When identical sequences were encoded by multiple genomic copies, CPM values were divided by copy number before downstream ranking. The CPM values at 24 and 72 h were shown as log10(CPM per copy + 1) in Figure 1b. Candidate Pol III promoters were required to be detected at 24 and 72 h, to have a minimum copy‐adjusted CPM of at least 100 across the two time points, and to remain within 1.5‐fold variation between 24 and 72 h. The retained tRNA, 5S rRNA and U6 snRNA candidates were selected.

To test the functional output of different Pol III promoters, each candidate promoter was used to drive the same *eGFP*‐targeting shRNA. 308‐eGFP strain without shRNA was used as the control. *eGFP* knockdown efficiency was calculated from the unit fluorescence intensity as follows:

$$Knockdown\;efficiency\;\left(\%\right)=\left[1-\frac{UFI_{shRNA}}{\bar{UFI_{eGFP\;control}}}\right]\times 100$$

where *UFI*_shRNA_ was the unit fluorescence intensity of the engineered strain carrying an *eGFP*‐targeting shRNA construct, and *UFI*_eGFP control_ with an overbar was the mean unit fluorescence intensity of the eGFP reporter control.

### 4.6 Pol III Promoter‐shRNA Library Composition

For testing the knockdown efficiency of different Pol III promoters, shRNA‐11 was used as the *eGFP* target position for all constructs. For testing the effect of target position on knockdown efficiency, the Pol III promoter tRNA‐Arg(CCT)‐1 was used to drive two test sets: one targeting 20 positions across *eGFP* and the other targeting the 13 genes shown in Figure 2. For testing the effect of promoter strength across different target genes, position 1 was used for each gene. The strong, medium and weak Pol III promoters were tRNA‐Gln(TTG)‐1, tRNA‐Glu(TTC)‐1 and tRNA‐Tyr(GTA)‐1, respectively.

For the *eGFP* dual‐control experiment, nine Pol III promoters were combined with six shRNA target positions, yielding 54 promoter–target position combinations. The promoters and target positions used are shown in Figure S3. For each of *CPT1* and *TGL* in Figure 3D, the dual‐control set comprised nine combinations of three promoters (strong, medium and weak) and three target positions (2, 3 and 4). In *Schizochytrium* sp., the RNAi libraries targeting the key genes in the β‐carotene biosynthesis pathway (*GGPPS*, *PSY* and *LCYB*) and the genes affecting the DHA/DPA ratio (*ORFC*, *PLD1* and *PEX10*) were constructed using the Pol III promoters listed in Table S3. For each target gene, the library contained 54 promoter‐target position combinations, consisting of nine Pol III promoters and six shRNA target positions. After screening, the engineered strains were amplified using the universal primers Sc‐shRNA‐TEST‐1 and Sc‐shRNA‐TEST‐2 located on the plasmid backbone, and the Pol III promoter and target‐site combination for each target gene was determined by Sanger sequencing (Sangon Biotech). The corresponding primer sequences are listed in Table S5.

In *Y. lipolytica*, six functional Pol III promoters with different strengths were used to construct the nine‐gene RNAi library. These included the previously reported SNR52 and tRNA_Gly_ promoters and three synthetic hybrid promoters (SCR1′–tRNA_Gly_, *RPR1*′–tRNA_Gly_ and SNR52′–tRNA_Gly_) [49], together with a 5S rRNA promoter. To establish RNAi in *Y. lipolytica*, we used the *Saccharomyces castellii Dcr1* and *Ago1* genes following the strategy reported by Fu et al. [39]. Their expression cassettes were introduced by chemical transformation and integrated at the InA‐1 locus of strain YIPDHA02 [47], generating YDHA. This strain information and gene sequences are provided in Tables S1 and S4, respectively. Further, for each target gene, the library contained 54 promoter‐target position combinations, consisting of six Pol III promoters and nine shRNA target positions. For comparing the knockdown efficiency of *C16E* and *Δ17D* in single‐gene and nine‐gene backgrounds, the tested promoter‐target position combinations consisted of three Pol III promoters (SCR1′‐tRNA_Gly_, tRNA_Gly_ and SNR52) and three shRNA target positions (2, 5, 8). After screening, the engineered strains were amplified using the universal primers yl‐shRNA‐TEST‐1 and yl‐shRNA‐TEST‐2 located on the plasmid backbone, and the Pol III promoter and target‐site combination for each target gene was determined by Sanger sequencing (Sangon Biotech). The detailed target position, Pol III promoter information and primer sequences are listed in Tables S2, S3 and S5.

### 4.7 RT‐qPCR Assay

For *Schizochytrium* sp., samples were collected at different fermentation times according to the assay. For testing the effect of fermentation time on knockdown efficiency, samples were collected at 24, 60, and 96 h. For all other RT‐qPCR assays, samples were collected at 60 h. Cells from 20 mL culture were collected by centrifugation at 12 000 × *g* in 4°C, and then ground in liquid nitrogen. A 50 mg sample was used for total RNA extraction with the FastPure Universal Plant Total RNA Isolation Kit (Vazyme Biotech, RC411). cDNA was synthesized using HiScript III RT SuperMix for qPCR (+gDNA wiper) (Vazyme Biotech, R323). RT‐qPCR was performed using Taq Pro Universal SYBR qPCR Master Mix (Vazyme Biotech, Q712). *β‐actin* was used as the internal reference gene for *Schizochytrium* sp. For *Y. lipolytica*, samples were collected at 48 h of fermentation, and total RNA extraction, cDNA synthesis and RT‐qPCR were performed using the same kits and procedures described above, with *G6PDH* as the internal reference gene. All RT‐qPCR primers used in this study were listed in Table S6.

Except for assays in which knockdown efficiency was calculated from unit fluorescence intensity, knockdown efficiency was calculated from relative transcript abundance as follows:

$$Knockdown\;efficiency\;\left(\%\right)=\left[1-\frac{R_{RNAi}}{\bar{R_{control}}}\right]\times 100$$

where *R*_RNAi_ was the relative transcript level of the target gene in the RNAi engineered strain, and *R*_control_ with an overbar was the mean relative transcript level of the corresponding control strain.

### 4.8 Lipid Extraction and Analysis

For lipid quantification and FA profile analysis, 50 mg lyophilized biomass was transferred to a reaction tube and mixed with 500 µL methanolic sodium hydroxide (1 mol L^−1^). An internal standard solution (100 µL) containing 2 g L^−1^ methyl heptadecanoate (Sigma–Aldrich) and 2 g L^−1^ glyceryl triheptadecanoate (Sigma–Aldrich) in hexane was added to each sample. The mixture was shaken at 200 × *g* for 5 h to release and derivatize fatty acids. The reaction was then neutralized with 40 µL 98% sulfuric acid. Fatty acid methyl esters were extracted with 400 µL hexane by shaking at 1200 rpm for 30 min. After centrifugation at 8000 × *g* for 3 min, the upper organic phase was collected into glass vials for gas chromatography.

FA methyl esters were measured on a GC‐2030 gas chromatography system (Shimadzu) equipped with a flame ionization detector and a DB‐23 column (60.0 m × 0.25 mm × 0.25 µm; Agilent). Nitrogen was used as the carrier gas at 20 mL min^−1^. The injector and detector were maintained at 250°C and 280°C, respectively. The injection volume was 1 µL. The oven program started at 100°C, increased to 196°C at 25°C min^−1^, then increased to 220°C at 2°C min^−1^ and was held for 10 min. Fatty acid methyl ester peaks were assigned by retention time. Total lipids and individual fatty acids were quantified using commercial fatty acid methyl ester standards and normalized to methyl heptadecanoate and glyceryl triheptadecanoate.

### 4.9 β‐Carotene Extraction and Analysis

Culture samples of *Schizochytrium* sp. (200 µL) were lyophilized for 48 h and mixed with 1 mL acetone and 0.2 g glass beads. The mixture was homogenized using a homogenizer and centrifuged at 12 000 × *g*. The supernatant was collected and passed through a 0.22 µm membrane filter for high‐performance liquid chromatography (HPLC).

β‐Carotene was measured on a 1260 Infinity II HPLC system (Agilent) equipped with an Eclipse XDB‐C18 reversed‐phase column (150 mm × 4.6 mm; Agilent). Mobile phase A consisted of methanol/water (10/90, v/v), and mobile phase B consisted of methanol/acetonitrile/dichloromethane (42/42/16, v/v/v). The gradient started at 20% A and 80% B, changed gradually to 0% A and 100% B over 0–15 min, and was held at this composition from 15 to 30 min. From 30 to 35 min, the composition was gradually returned to 20% A and 80% B. The flow rate was maintained at 1 mL min^−1^, and β‐carotene was detected at 450 nm. A β‐carotene reference standard was purchased from Aladdin.

### 4.10 5 L Fed‐Batch Fermentation

Fed‐batch fermentation was performed in a 5 L bioreactor (T&J Bioengineering) with a working volume of 3 L. The bioreactor was inoculated with 10% seed culture based on biomass. Aeration was maintained at 4 vvm, and the agitation speed was set to 450 rpm. The pH was controlled at 6.5 using NaOH or citric acid. The initial glucose concentration was 70 g L^−1^. During fermentation, residual glucose was measured every 12 h. When the glucose concentration decreased below 50 g/L, feeding solution was added to restore the glucose concentration to 30–50 g L^−1^.

### 4.11 Strain Stability Assay

For the strain stability assay, single colony was isolated from strains maintained on seed agar medium and inoculated into 50 mL seed liquid medium. Every 24 h, 1 mL culture was transferred into fresh seed liquid medium for continuous passaging until P20. Samples from P5, P10, P15, and P20 were spread onto seed agar medium to obtain single colony. Eight colonies were randomly selected from each sampled passage and cultured in shake flasks as described above. The DHA/DPA ratio and lipid titer were analyzed using a GC‐2030 system (Shimadzu). RT‐qPCR was performed for the three target genes to determine whether their relative transcript abundance changed during passaging (Figure S8).

### 4.12 Statistics Analysis

All experiments were performed with three biologically independent replicates, each representing an independent transformant, and data were presented as the mean ± s.d. Statistical analyses were performed using two‐sided Welch's *t*‐tests. For multiple comparisons, each experimental group was compared with the corresponding control using two‐sided Welch's *t*‐tests, followed by Holm correction. Significance was defined as follows: ns, not significant; *p < 0.05; **p < 0.01; ***p < 0.001.

## Author Contributions

Conceptualization: **Z.X.Z**. and **X.M.S**. Methodology: **Z.X.Z**., **Y.S.X**., and **F.D**. Investigation: **Z.X.Z**., **Y.S.X**., and **F.D**. Validation: **Z.X.Z**., **Y.S.X**., and **F.D**. Formal analysis: **Z.X.Z**., **Z.J.L**., **W.M**., and **G.Y**. Data curation: **Z.X.Z**., **Z.J.L**., **W.M**., and **G.Y**. Writing – original draft: **Z.X.Z**. and **Y.S.X**. Writing – review and editing: **Z.X.Z**., **Y.S.X**., and **X.M.S**. Supervision: **X.M.S**. Project administration: **X.M.S**. All authors read and approved the final manuscript.

## Conflicts of Interest

The authors declare no conflicts of interest.

## Supporting information




**Supporting File**: advs78608‐sup‐0001‐SuppMat.docx.

## Data Availability

The data that support the findings of this study are available from the corresponding author upon reasonable request.
