# Supplementary material for: Combinatorial Tuning of Multigene Biosynthetic Pathways based on RNA Interference
Source: Adv Sci (Weinh). 2026 Nov 3:e78608. Online ahead of print. doi: 10.1002/advs.78608 (PMC13731073; doi:10.1002/advs.78608)
Supplement: Supplementary file 1 [file ADVS-9999-e78608-s001.docx]

**Combinatorial Tuning of Multigene Biosynthetic Pathways based on RNA interference**

Zixu Zhang^1,2,3^, Yingshuang Xu^1,2,3^, Zijia Li^1,2^, Fei Du^1,2^, Wang Ma^1,2^, Guang Yang^1,2^, Xiaoman Sun^1,2^*

^1^State Key Laboratory of Microbial Technology, Nanjing Normal University, Nanjing, 210046, People’s Republic of China.

^2^School of Food Science and Pharmaceutical Engineering, Nanjing Normal University, Nanjing, 210046, People’s Republic of China.

^3^These authors contributed equally.

*Correspondence: [xiaomansun@njnu.edu.cn](mailto:xiaomansun@njnu.edu.cn)

**Supplementary Figure 1. Ranking of RNA polymerase III-transcribed small RNAs by transcriptional level at 24 h and 72 h. A,** tRNA. **B,** 5S rRNA. **C,** U6 snRNA. CPM, counts per million.

**Supplementary Figure 2. Relationship between relative eGFP mRNA level and knockdown efficiency.**

**Supplementary Figure 3. eGFP fluorescence matrix generated by promoter-position dual control.**

**Supplementary Figure 4. β-Carotene titers of the top10 strains.**

**Supplementary Figure 5. DHA/DPA ratios of WT and knocking down of ORFC, PLD1, and PEX10.**

**Supplementary Figure 6. Combinatorial RNAi tuning scheme for the top ten LDPA strains.** Promoter strength and target-position combinations used to tune ORFC, PEX10 and PLD1 in the top ten engineered LDPA strains.

**Supplementary Figure 7. Lipid titers of the top10 LDPA strains.**

**Supplementary Figure 8. Stability of knockdown efficiency for target genes affecting the DHA/DPA ratio.** Points indicate biological replicate measurements; center lines indicate the mean.

**Supplementary Table 1. Main strains and plasmids used in this study.**

| **Strains** | **Characteristics** | **References** |
| --- | --- | --- |
| *Schizochytrium* sp. HX-308 | Wild-type strain, CCTCC M 209059 | Lab stock |
| *A. tumefaciens* AGL1 | C58, recA::*bla*, pTiBo542ΔT-DNA, Rif, Cb | Lab stock |
| *E.coli* DH5α | *F-, φ80 dlacZ ΔM15, Δ (lacZYA –argF) U169, deoR, recA1, endA1,  hsdR17(rK-, mK+), phoA, supE44, λ-, thi -1, gyrA96, relA1* | Tsingke |
| YIPDHA02 | *Y. lipolytica* Po1f derivate (*matA, xpr2-322, axp2-delta NU49, XPR2::SUC2, Ura-, LEU-, Ku70-*),  *PEX10::P_TEF_-IgΔ9E-T_CYC1T_-P_EXP_-CcΔ8D-T_XPR2T_-P_hp8d_-MaΔ5D-T_Mig1T_-P_YAT_-PaΔ17D-T_Lip2T_-P_FBAin_-PaΔ5E-T_Lip1T_-P_GPD_-IsΔ4D-T_XPR2T_* | Lab stock |
| 308-eGFP | *Schizochytrium* sp. HX-308 derivate,  Int1::*P_2902_-eGFP-T_CYC1t_* | This study |
| 308-eGFP-RdRP | 308-eGFP derivate,  Int2::*P_2902_-RdRP-T_CYC1t_* | This study |
| 308-RdRP | *Schizochytrium* sp. HX-308 derivate,  Int1::*P_2902_-RdRP-T_CYC1t_* | This study |
| POA01 | 308-RdRP derivate,  Int2::*P_3626_-SsΔ9D-T_CYC1t_* | This study |
| POA02 | POA01 derivate,  Int3::*P_tRNA-Arg(CCT)-1_-shRNA_TGL_-1-T* | This study |
| POA03 | POA01 derivate,  Int3::*P_tRNA-Gln(TTG)-1_-shRNA_TGL_-4-T* | This study |
| Car-OE | 308-RdRP derivate,  Int2::*P_2902_-GGPPYS-T_CYC1t_-P_3626_-PSY-T_CYC1t_- P_2902_-LCYB-T_CYC1t_*; Int3:: *P_2902_-GGPPYS-T_CYC1t_-P_3626_-PSY-T_CYC1t_- P_2902_-LCYB-T_CYC1t_* | This study |
| Car-01 | Car-OE derivate,  Int4::*P**_tRNA-Arg(CCT)-1_-shRNA_GGPPS_-1-T- P_tRNA-Tyr(GTA)-1_ -shRNA_PSY_-3-T-P_tRNA-Gly(GCC)-1_ -shRNALCYB-3-T* | This study |
| YDHA | YIPDHA02 derivate, *InA-1*:: *P_TEF_-* *Dcr1-T_CYC1T_-P_EXP_-* *Argo1-T_XPR2T_* | This study |
| YDHAi01 | YDHA derivate,  XPR2::*P_tRNAGly_-shRNA_ylC16E_-4-T-P_SNR52_-shRNA_ylΔ9D_-6-T-P_5S rRNA_-shRNA_ylΔ12D_-1-T-P_RPR1′–tRNAGly_-shRNA_IgΔ9E_-7-T-P_SCR1′-tRNAGly_-shRNA* *_CcΔ8D_-3-T-P_SNR52′–tRNAGly_-shRNA_MaΔ5D_-9-T-P_SNR52_-shRNA_PaΔ17D_-2-T-P* *_SNR52′–tRNAGly_-shRNA_PaΔ5E_-5-T-P_SNR52_-shRNA_IsΔ4D_-1-T* | This study |
| YDHAi01-2copy | YDHAi01 derivate,  InTF3:: *P_ylC16E_-ylC16E-T_ylC16E_-P_ylΔ9D_-ylΔ9D-T_Δ9D_-P_ylΔ12D_-ylΔ12D-T_ylΔ12D_- P_TEF_-IgΔ9E-T_CYC1T-_P_EXP_-CcΔ8D-T_XPR2T_-P_hp8d_-MaΔ5D-T_Mig1T_-P_YAT_-PaΔ17D-T_Lip2T_-P_FBAin_-PaΔ5E-T_Lip1T_-P_GPD_-IsΔ4D-T_XPR2T_* | This study |
| YDHAi01-3copy | YDHAi01-2copy derivate,  *InTC*:: *P_ylC16E_-ylC16E-T_ylC16E_-P_ylΔ9D_-ylΔ9D-T_Δ9D_-P_ylΔ12D_-ylΔ12D-T_ylΔ12D_- P_TEF_-IgΔ9E-T_CYC1T-_P_EXP_-CcΔ8D-T_XPR2T_-P_hp8d_-MaΔ5D-T_Mig1T_-P_YAT_-PaΔ17D-T_Lip2T_-P_FBAin_-PaΔ5E-T_Lip1T_-P_GPD_-IsΔ4D-T_XPR2T_* | This study |
| YIPDHA02-2copy | YIPDHA02 derivate,  InTF3:: *P_ylC16E_-ylC16E-T_ylC16E_-P_ylΔ9D_-ylΔ9D-T_Δ9D_-P_ylΔ12D_-ylΔ12D-T_ylΔ12D_- P_TEF_-IgΔ9E-T_CYC1T-_P_EXP_-CcΔ8D-T_XPR2T_-P_hp8d_-MaΔ5D-T_Mig1T_-P_YAT_-PaΔ17D-T_Lip2T_-P_FBAin_-PaΔ5E-T_Lip1T_-P_GPD_-IsΔ4D-T_XPR2T_* | This study |
| YIPDHA02-3copy | YIPDHA02-2copy derivate,  *InTC*:: *P_ylC16E_-ylC16E-T_ylC16E_-P_ylΔ9D_-ylΔ9D-T_Δ9D_-P_ylΔ12D_-ylΔ12D-T_ylΔ12D_- P_TEF_-IgΔ9E-T_CYC1T-_P_EXP_-CcΔ8D-T_XPR2T_-P_hp8d_-MaΔ5D-T_Mig1T_-P_YAT_-PaΔ17D-T_Lip2T_-P_FBAin_-PaΔ5E-T_Lip1T_-P_GPD_-IsΔ4D-T_XPR2T_* | This study |
| LDPA01 | 308-RdRP derivate,  Int2::*P_tRNA-Gly(GCC)-1_-shRNA_ORFC_-4-T-_tRNA-Gly(TCC)-1_-shRNA**_PEX10_-5-T-P_5S rRNA-7_-shRNA _PLD1_-3-T* | This study |
| LDPA01-T | LDPA01 derivate,  Int3:: *P_tRNA-Gln(TTG)-1_-shRNA_TGL_-4-T* | This study |
| LDPA01-A | LDPA01 derivate,  Int3:: *P_tRNA-Arg(CCT)-1_-shRNA_ACOX1_-1-T* | This study |
| LDPA01-TA | LDPA01-T derivate,  Int4:: *P_tRNA-Arg(CCT)-1_-shRNA_ACOX1_-1-T* | This study |
| **Plasmids** | **Characteristics** | **References** |
| pZPK-G418 | pZPK backbone, G418 resistance cassette, Kan, used for genome integration at Int1 | Lab stock |
| pZPK-Nourse | pZPK backbone, nourseothricin resistance cassette, Kan, used for genome integration at Int2 | Lab stock |
| pZPK-Zeo | pZPK backbone, zeocin resistance cassette, Kan, used for genome integration at Int3 | Lab stock |
| pZPK-Hyg | pZPK backbone, hygromycin B resistance cassette, Kan, used for genome integration at Int4 | Lab stock |
| pUC-57 | ColE1 Ori, LacZ, Amp | Lab stock |
| pUC-HUH | ColE1 Ori, Amp, His-URA-His | Lab stock |
| pZPK-G418-eGFP | pZPK-G418 derivative, Int1::*P_2902_-eGFP-T_CYC1t_* | This study |
| pZPK-G418-RdRP | pZPK-G418 derivative, Int1::*P_2902_-RdRP-T_CYC1t_* | This study |
| pZPK-Nourse-RdRP | pZPK-Nourse derivative, Int2::*P_2902_-RdRP-T_CYC1t_* | This study |
| pZPK-Nourse-SsΔ9D | pZPK-Nourse derivative, Int2::*P_3626_-SsΔ9D-T_CYC1t_* | This study |
| pZPK-Zeo-shTGL-1 | pZPK-Zeo derivative, Int3::*P_tRNA-Arg(CCT)-1_-shRNA_TGL_-1-T* | This study |
| pZPK-Zeo-shTGL-4 | pZPK-Zeo derivative, Int3::*P _tRNA-Gln(TTG)-1_-shRNA_TGL_-4-T* | This study |
| pZPK-Nourse-Car-OE | pZPK-Nourse derivative, Int2::*P_2902_-GGPPYS-T_CYC1t_-P_3626_-PSY-T_CYC1t_- P_2902_-LCYB-T_CYC1t_* | This study |
| pZPK-Zeo-Car-OE | pZPK-Zeo derivative, Int3::*P_2902_-GGPPYS-T_CYC1t_-P_3626_-PSY-T_CYC1t_- P_2902_-LCYB-T_CYC1t_* | This study |
| pZPK-Hyg-shRNA-Car01 | pZPK-Hyg derivative, Int4::*P_tRNA-Arg(CCT)-1_-shRNA_GGPPS_-1-T- P_tRNA-Tyr(GTA)-1_ -shRNA_PSY_-3-T-P_tRNA-Gly(GCC)-1_ -shRNA_LCYB_-3-T* | This study |
| pUC-HUH- YDHA | pUC-HUH derivative, *InA-1*:: *P_TEF_-* *Dcr1-T_CYC1T_-P_EXP_-* *Argo1-T_XPR2T_* | This study |
| pUC-HUH-YDHAi01 | pUC-HUH derivative, XPR2::*P_tRNAGly_-shRNA_ylC16E_-4-T-P_SNR52_-shRNA_ylΔ9D_-6-T-P_5S rRNA_-shRNA_ylΔ12D_-1-T-P_RPR1′–tRNAGly_-shRNA_IgΔ9E_-7-T-P_SCR1′-tRNAGly_-shRNA* *_CcΔ8D_-3-T-P_SNR52′–tRNAGly_-shRNA_MaΔ5D_-9-T-P_SNR52_-shRNA_PaΔ17D_-2-T-P* *_SNR52′–tRNAGly_-shRNA_PaΔ5E_-5-T-P_SNR52_-shRNA_IsΔ4D_-1-T* | This study |
| pUC-HUH- DHA -2copy | pUC-HUH derivative, InTF3:: *P_ylC16E_-ylC16E-T_ylC16E_-P_ylΔ9D_-ylΔ9D-T_Δ9D_-P_ylΔ12D_-ylΔ12D-T_ylΔ12D_- P_TEF_-IgΔ9E-T_CYC1T-_P_EXP_-CcΔ8D-T_XPR2T_-P_hp8d_-MaΔ5D-T_Mig1T_-P_YAT_-PaΔ17D-T_Lip2T_-P_FBAin_-PaΔ5E-T_Lip1T_-P_GPD_-IsΔ4D-T_XPR2T_* | This study |
| pUC-HUH-DHA-3copy | pUC-HUH derivative, *InTC*:: *P_ylC16E_-ylC16E-T_ylC16E_-P_ylΔ9D_-ylΔ9D-T_Δ9D_-P_ylΔ12D_-ylΔ12D-T_ylΔ12D_- P_TEF_-IgΔ9E-T_CYC1T-_P_EXP_-CcΔ8D-T_XPR2T_-P_hp8d_-MaΔ5D-T_Mig1T_-P_YAT_-PaΔ17D-T_Lip2T_-P_FBAin_-PaΔ5E-T_Lip1T_-P_GPD_-IsΔ4D-T_XPR2T_* | This study |
| pZPK-Nourse-shRNA-LDPA01 | pZPK-Nourse derivative, Int2::*P_tRNA-Gly(GCC)-1_-shRNA_ORFC_-4-T-_tRNA-Gly(TCC)-1_-shRNA_PEX10_-5-T-P_5S rRNA-7_-shRNA _PLD1_-3-T* | This study |
| pZPK-Zeo-shRNA-LDPA01-T | pZPK-Zeo derivative, Int3::PtRNA-Gln(TTG)-1-shRNATGL-4-T | This study |
| pZPK-Zeo-shRNA-ACOX1 | pZPK-Zeo derivative, Int3:: *P_tRNA-Arg(CCT)-1_-shRNA_ACOX1_-1-T* | This study |
| pZPK-Hyg- shRNA-ACOX1 | pZPK-Hyg derivative, Int4:: *P_tRNA-Arg(CCT)-1_-shRNA_ACOX1_-1-T* | This study |

**Supplementary Table 2. 21bp shRNA target sequences used in this study**.

| **Gene** | **Position** | **21bp shRNA target sequence (5'→3')** |
| --- | --- | --- |
| ***Schizochytrium* sp.** | | |
| eGFP | 1 | ATGGTGAGCAAGGGCGAGGAG |
|  | 2 | TGCCCATCCTGGTCGAGCTGG |
|  | 3 | CCACAAGTTCAGCGTGTCCGG |
|  | 4 | TGCCACCTACGGCAAGCTGAC |
|  | 5 | ACCACCGGCAAGCTGCCCGTG |
|  | 6 | TGACCACCCTGACCTACGGCG |
|  | 7 | CCCGACCACATGAAGCAGCAC |
|  | 8 | TCCGCCATGCCCGAAGGCTAC |
|  | 9 | ATCTTCTTCAAGGACGACGGC |
|  | 10 | CCGAGGTGAAGTTCGAGGGCG |
|  | 11 | CATCGAGCTGAAGGGCATCGA |
|  | 12 | GGGCACAAGCTGGAGTACAAC |
|  | 13 | AGCCACAACGTCTATATCATG |
|  | 14 | ACGGCATCAAGGTGAACTTCA |
|  | 15 | CGAGGACGGCAGCGTGCAGCT |
|  | 16 | ACCACTACCAGCAGAACACCC |
|  | 17 | CCGACAACCACTACCTGAGCA |
|  | 18 | CCCAACGAGAAGCGCGATCAC |
|  | 19 | GCTGGAGTTCGTGACCGCCGC |
|  | 20 | ATGGACGAGCTGTACAAGTAA |
| ORFA | 1 | GTTCTACTCGCGCCTTAATTA |
|  | 2 | AAAGACAAAGGGCATGCTCAT |
|  | 3 | ATGAGATCCACGCTGTTATTC |
| ORFB | 1 | AACTCCTCAACGTGTACCAAA |
|  | 2 | TATCGATGACGAGACCAAGAT |
|  | 3 | CATTGAAGATGTTGAGGTCGA |
| ORFC | 1 | CATCTCCATGTTCTTCTTCGA |
|  | 2 | TGCCGGAAATCAACTACAAAC |
|  | 3 | AAGATCGTCGTCGACTTTAAG |
|  | 4 | CAAGACGGTCAACCCGAACGA |
|  | 5 | CTACGCCGTCAACCTCATCCA |
|  | 6 | CTTCATCGTCACCGGCACTGT |
| PPTase | 1 | CCTTCAGCTTTAATCTTTCGC |
|  | 2 | TGGGTGTGGACATTATGAAGT |
|  | 3 | ACACTTGCAACGCTTCTACAA |
| MCAT | 1 | TGGCCAATGCTAATAGTCCTA |
|  | 2 | CTGTGGAAATTGCCAAGGAAA |
|  | 3 | TTTACGGCAAGGATAAGGAAG |
| FAS | 1 | TCATCTTCCTCACCCAAATGT |
|  | 2 | CATCTCCATCAACATGCTCTA |
|  | 3 | GATCGAGTTCAAGGTCTTTGA |
| C16E | 1 | CGACATGATCAAGGACATGAA |
|  | 2 | GGGAGACCATGAAATTTGACA |
|  | 3 | CTTTGCCTACCTCGTCATTAT |
| C18E | 1 | AGGCCAATAAGATGAATGACG |
|  | 2 | TCCGTACATTACCACGATTCA |
|  | 3 | TTTGAGCTCAAGTACCTCAAG |
| C18/C20E | 1 | TAAATTCGGCGGCAACATCAA |
|  | 2 | TCTCATCATCAAAGGCAAAGG |
|  | 3 | TGTCTACTACTTTACCAACGC |
| C20/C22E | 1 | TCTCATTCGAACTGTTCCTCA |
|  | 2 | TAGGTCTGTGGTCTTTCTTGT |
|  | 3 | TACGGTCTTTATTGTCCTTCG |
| △12D | 1 | CGTCTTTGTTTATCTCCTCCT |
|  | 2 | CATTATCCATGGCAAGCACAA |
|  | 3 | CAGCCTACGTAATTGTTCAAG |
| △4D | 1 | GTTTCCAGCACATTTATGGAC |
|  | 2 | TATCAACAAGGTCGTCACTCA |
|  | 3 | AAGCTCTTTGCTATTGCGCAT |
| △5D | 1 | TGTTGTTCTCTTCGTGCTTTC |
|  | 2 | GCTGTGCTTCATCCAATCAAT |
|  | 3 | ATACTACGTCAGCGACTCAAA |
| △8D | 1 | TCTGGTTTGGGTTCTTTGTCT |
|  | 2 | GCTATCAGCACATTCTCTACT |
|  | 3 | ATATTGTTCTTTCCTACGCCG |
| ACC1 | 1 | GAACAACTACAACAACGTCAG |
|  | 2 | TGACAAGATCATGAGCAGCAT |
|  | 3 | GACAACGACAACTTCTTCTTC |
| ACSS | 1 | AAGAGAACAAGCGCAAGAAGA |
|  | 2 | ACCTGCAAGATCATCTTCGAT |
|  | 3 | CTACATGAACACCTACCTCAA |
| PDH | 1 | AACGAGAACGACAAGATGAAC |
|  | 2 | CGAATACTACAAGCAGGGTAA |
|  | 3 | ATGCACATGTACAACAAGTCC |
| G6PD | 1 | CTTCCTCGACATTTGCATCTA |
|  | 2 | TCGAGGAATTCAAGGAGACAA |
|  | 3 | GCCTCTATTTAATCGTGACCA |
| 6PGD | 1 | CAAATTCCTTCACCGCATCAA |
|  | 2 | GTAATGAGTGGTTCGAGAACT |
|  | 3 | TCATCAAGCCCATCATCGAAA |
| IDH | 1 | TCAACAAGGGCAACATCATGA |
|  | 2 | CAACGGCAAGTACAAGAAGAT |
|  | 3 | TCGTCGACATGAACATCTTTC |
| HMGS | 1 | TACCGAGACCATCATTGACAA |
|  | 2 | ACATCAACAGCATGTGCATGT |
|  | 3 | CTACAACCTGTACACCAAGAA |
| HMGCR | 1 | TTCTTGGCTTCAACAGCACTA |
|  | 2 | AAAGCTCAAGGACATCTTTGC |
|  | 3 | AAAGGTCCTCAAGACCAACAT |
| MVK | 1 | AGGTTGACGAGGATGAGATTA |
|  | 2 | AATACATGGAGGTCTTTGACG |
|  | 3 | CACTTTGTCGTGTACAAGAAG |
| IDI | 1 | GATGACAAGATCACCTTTCCT |
|  | 2 | CATTGAGAAGTTCCACTACCT |
|  | 3 | TCATTTCTCCTTGGTTCGGAT |
| ERG20 | 1 | ATCTGATGGATTCGTCCATCA |
|  | 2 | CTTGCGATATTTGCGTCAAGA |
|  | 3 | CATTAAGACTGTCAAGGGCAT |
| GGPPS | 1 | CTTGGCATGATCATGGATAAG |
|  | 2 | TCGTCAATCTGCTTTCCATCT |
|  | 3 | TACCTCTTGTCTATCCTCAAG |
| SQS | 1 | CAAATCCAGGAAATCAAGCAC |
|  | 2 | TTTATATTGTCCTTCGCGCAC |
|  | 3 | TCAAAGCAATTACCTTGCCTG |
| GPAT1 | 1 | CTGTTGCGGAAGAAGATGAAA |
|  | 2 | AAATGACGATGACGATGACAG |
|  | 3 | AAAGAGTCAATCCACCCAAGA |
| GPAT2 | 1 | TGCTGTGCTTTAATCATGGCA |
|  | 2 | ACGGCCAATCTAATTGAGTCT |
|  | 3 | CCATGGGAGTTCTATTTAACG |
| LPAT1 | 1 | ATACGTCGTACATTGATCCCT |
|  | 2 | ATGGTCTGCCGAACTTTAACT |
|  | 3 | CGTGCACTTTGTAAAGGACAA |
| LPIN | 1 | TCATCAACGACACACGATCAT |
|  | 2 | AACTTTAACGTTCGCTTCACG |
|  | 3 | TTTGCTTCCTCTACAATCACC |
| DGAT1 | 1 | GCATTGTGAGCAGGATCTTTA |
|  | 2 | TTACGACCGCCATAGACTTTA |
|  | 3 | CATTATTGGTGATGGTGTGAG |
| DGAT2 | 1 | CGCTGCTACTATTCATCTTTG |
|  | 2 | GTTGTCATTGGCATTGTGCTA |
|  | 3 | ATCTTTGGTATCCTTTCGCTC |
| DGAT3 | 1 | ACTCAATCGGATCGAAACCAA |
|  | 2 | GTGGATCATTTACGTGGACAA |
|  | 3 | CAAAGAAGTCCTGCAACCAAA |
| MGAT2 | 1 | GATGAAGAGGAAGAAGATGCA |
|  | 2 | CAAGAAGAGATGAGCATTGAC |
|  | 3 | TCCCGTGAATTTGAAGACAGA |
| TGL | 1 | GTGATTCTTGTGGGTCATTCA |
|  | 2 | CGATGAACTGGTCAAATCTCT |
|  | 3 | ATTCGCGCTCTTGAATGTCTA |
|  | 4 | TGCGGAGGCCATCAAGGCAAG |
| DGL | 1 | TGAGCAAGATGCAGAGAATGA |
|  | 2 | GTTCACTACTCTTACCATCAG |
|  | 3 | CAGGGCGCTTATTATGTTGAT |
| MGL | 1 | TTTCACTGGCATGGATTTGCA |
|  | 2 | CACGTGACTGATCTTGTACAA |
|  | 3 | TCAAGAAGAGACCCATCAACA |
| LIPA | 1 | GAGGAAGAAGGACAACAAGAA |
|  | 2 | TACAACGATTACCACGACGAA |
|  | 3 | CACAGCAATAGCAGCACAAAT |
| PLD1 | 1 | GACGCATTATGATTCCTCAGT |
|  | 2 | GTCCTTTGTTGCCTTCTTTGA |
|  | 3 | CGGTGCGAATTAAATTTCCTG |
|  | 4 | GCATCAATCGAGGACCAAACT |
|  | 5 | ATGGCAGCAATGGTGAAGACT |
|  | 6 | GGTGACGAGGTCTTCATCTAG |
| PGC1 | 1 | GTATGGCTCCAAGATTGTTGT |
|  | 2 | CTTTGACGCCGATATCATCAC |
|  | 3 | CTTGAAGACCAAGACGACGAT |
| CPT1 | 1 | TGCCATCAACTCGAACTACTA |
|  | 2 | TTTGCGCTTCAAGGATCTTGT |
|  | 3 | AAATGATTGTCAAGCACGCGA |
|  | 4 | CCGGCAATTGCGCACCTGTGG |
| CPT2 | 1 | TACAGCTACATTGAGCAGTTC |
|  | 2 | TTGACAAGCACGTCATTATCC |
|  | 3 | ATGAGGAGTTCGAGAACACCA |
| CROT | 1 | ACATTAATTGCTTCCAGACCC |
|  | 2 | CTCATCGCCATCAAGAATGAT |
|  | 3 | CACTCTTTCACGTCGTTCTTA |
| PEX10 | 1 | AAAGGACGCGTACTATAGACA |
|  | 2 | TGGAATTACGAGCAACGATCA |
|  | 3 | GGAATGGAGCATATCAAGTGT |
|  | 4 | TGCGTCTATTTCACCTTGTGA |
|  | 5 | TTCTTGACGCCGATGAAATTG |
|  | 6 | ATGGCGAGTCCCATGCAGCAG |
| ACOX1 | 1 | GAAGATAAACTCGAACTCCTC |
|  | 2 | TAAAGCAGCCACACAATGGAT |
|  | 3 | AGGTCAATCTTGATGACCTTC |
| ACOX2 | 1 | ATTGCCGACCTCATTGTCAAT |
|  | 2 | AATCATCTTTGGTTCTTCGCC |
|  | 3 | AAACTGGATTAGCCAAGGTCT |
| ACADM | 1 | GGCGACAAGTACATTCTCAAT |
|  | 2 | CTACAAGTCTGCCATGATCAT |
|  | 3 | CAAGGAGATTAACATGGGCCA |
| ECHS1 | 1 | GCGACAATCCGATTCAATGAT |
|  | 2 | ATTATTGAAGAGCTTTGCGGC |
|  | 3 | CCTTTGTGCAATTGGGTTTAC |
| LCAD | 1 | AATCGTCAGAATGACCTCCAA |
|  | 2 | AGAAATCAACGATGGCGTCAA |
|  | 3 | TCTCTCTTTATCGTACCCAAG |
| GGPPS | 1 | TTTCCACGAGACCGACGCTCA |
|  | 2 | CTCGGTCGCCGCTCTTGGGCA |
|  | 3 | CTGCGTCGCGGGCAACCCGTA |
|  | 4 | ATGATCATGGATAAGACGGGT |
|  | 5 | AAGAACTCGGAGCCAGGAGAT |
|  | 6 | GTTGACGACTGCGATATTTGA |
| PSY | 1 | ATGGCGCGCATGGGCGTCAGC |
|  | 2 | GAGGTCCGAAAGGGCGATCGA |
|  | 3 | TCTCAATATGGTCCGCGCGGT |
|  | 4 | GCGAGTCACGCCGGCAAAGCC |
|  | 5 | CAAGCGCTCACAGAGACTGTG |
|  | 6 | AACTTCCTAAAGACCTTCTAG |
| LCYB | 1 | TCGTCGTCGTCCTCGTCGTCG |
|  | 2 | CCCTGCGTTGTATTCCATGAT |
|  | 3 | CGTGCTCAAACCCTCCATGGA |
|  | 4 | TTGGCGAGGATAATCGCTTGG |
|  | 5 | TTTCAGCCCACGACGTTGCAG |
|  | 6 | CCGCTGTACAAGAATGCTTGA |
| ***Yarrowia lipolytica*** | | |
| yl16E | 1 | ATGCTCTCGTCAATCTCGCCC |
|  | 2 | GGATGTACAATGCCTCTCTGG |
|  | 3 | TTGCCTTTGCCAAGACCGCAC |
|  | 4 | CTACCAAATCACAGGAGACAG |
|  | 5 | TTCTATCTGTCCAAATTCTAC |
|  | 6 | GGGCCGGCATCCGATTCGCCT |
|  | 7 | TCCCAAGATCCTCAAGGCATC |
|  | 8 | GACAGTCTGTTCTTGTCTCAC |
|  | 9 | TTGAAGGCCAAGTCCAACTAG |
| ylΔ9D | 1 | AAACGTGGACCAAGTGGATCT |
|  | 2 | CAACTGGCTCAACTTCATTCT |
|  | 3 | TCTGCCCGTTCGAATCATCCT |
|  | 4 | CCAAGAACAAGGGCCGAACTG |
|  | 5 | ACCTTTGTGCAGCAGGTGACT |
|  | 6 | CATCTGGTACCAGTACGACCC |
|  | 7 | GTCATTGAGTTTGAGGAGTTC |
|  | 8 | CCAACGCTGGCCACAACCTGC |
|  | 9 | ATGTCTGGCATGGCTGCTTAG |
| ylΔ12D | 1 | ATGGCCTCAACGAGTGCACTG |
|  | 2 | GAGTACGGCGTCATGCTTGAC |
|  | 3 | CTTCGTAACTCCCGAGTACAT |
|  | 4 | GTTCGCTGCTGGTGCCTTACT |
|  | 5 | CCTCCTTATGCTCGTGCTGCA |
|  | 6 | ATGCCAAGCTGATTGTGCTCT |
|  | 7 | CTCACTACACCAACGACGAGT |
|  | 8 | CCCATCATGGGCAAGCATTAC |
|  | 9 | GTTATCAAGCCTGTTGCTTGA |
| IgΔ9E | 1 | ATGGCCACTGAAGCGACTGCA |
|  | 2 | CGGTCATCTGGTCTGGTAGAC |
|  | 3 | TTACGTCACGGCTACAGCACT |
|  | 4 | TAGCCGCGTCTGGGACTCGAA |
|  | 5 | TGAAGGGTAAGAACGTGTCCT |
|  | 6 | AGGGCGTGTGGATCTTCATGT |
|  | 7 | TCACAGCCATGCAGATTTCTC |
|  | 8 | TTCTCCTGGGTCTTCAACTAC |
|  | 9 | AAGGACAATCTGGCATCGAAA |
| CcΔ8D | 1 | ATGGGTGTGAAGAAGGCTGAC |
|  | 2 | TCGGCTGCGCTTACAAGGCTG |
|  | 3 | ACGAGTGCGGCCACCAGGCCT |
|  | 4 | CACACCCGATCCGAGCTGGGC |
|  | 5 | CGCTTACATCCTGATGAACGC |
|  | 6 | TTGCATCCATTAACCACTGGG |
|  | 7 | ACCGATCTCTGCTCGGCGACC |
|  | 8 | ACCGAGGGACGACCCGGAGGA |
|  | 9 | GGCATCGAGGTGGAGAAGTAG |
| MaΔ5D | 1 | ATGGGCACCGACCAGGGAAAG |
|  | 2 | CCCGTCTTTGAGATGTACCAC |
|  | 3 | AACATGGACTCCAAGAACCGA |
|  | 4 | GGCCTGAACCCTCTCCACGAC |
|  | 5 | GACCCTGACGTCTCTACTTCT |
|  | 6 | AACGACGCCATCCGAGTGAAC |
|  | 7 | CTCGCCCTCACCTTCCAGGCC |
|  | 8 | TACCAGGCCGTGCACCACCTC |
|  | 9 | CTCCGACCTAAGGAGGAGTAA |
| PaΔ17D | 1 | ATGGCTTCCTCTACCGTTGCC |
|  | 2 | GCCGGTTCCCTTGCTCTCGGA |
|  | 3 | TCGGTCACGACTGTGGACATG |
|  | 4 | ACACCGGCAACATCGACAAGG |
|  | 5 | GAAAGGTCAACCACTTCAATC |
|  | 6 | CATGGCTCTGTATTACTTCGC |
|  | 7 | GTCCTCTGTGGACCGAAGCTA |
|  | 8 | GCCTTTCCCGAACTGGTGCGA |
|  | 9 | GCCAAGACCAAAGCCAACTAA |
| PaΔ5E | 1 | ATGGCTGCTCGAGTAGAGAAG |
|  | 2 | GCTCGGCCTTCGCTTATGCAT |
|  | 3 | CCCTTATGTATGTCATCGCCG |
|  | 4 | GATTGTTGTATGCTCATGGAT |
|  | 5 | GTCCATCTGCTAACCAAGTTC |
|  | 6 | ACCATTGGAATGGTGTGGTCT |
|  | 7 | CATTAACAATCCTTTCAAACG |
|  | 8 | CGCGTACCTGCAGATCTCCTA |
|  | 9 | ACTTCCAAGAAGGCCCAGTGA |
| IsΔ4D | 1 | ATGTGTAACGCCGCCGTGGAG |
|  | 2 | ACGGCCACCACAAGGGCGCCT |
|  | 3 | CTCTGCCGTCTACGGCATTTG |
|  | 4 | TGGTCCTTCGCCTTTCAGCAC |
|  | 5 | CACCCTCGCCAAGAAGGTCCC |
|  | 6 | ACTACGCCATCTTTGCCTGGG |
|  | 7 | GTCTACTATGATCGGCACCAT |
|  | 8 | AGTTTGGACTGCCCTACCGAG |
|  | 9 | GAGACTCTGAAGGCTGATTAA |

For *GGPPS*, target-position numbering is specific to each experiment. The first three entries belong to the candidate-gene screening set, and the six entries listed with PSY and LCYB belong to the carotenoid-pathway library.

**Supplementary Table 3. Pol III promoter sequences used for shRNA expression in this study**

| **Promoter** | **Sequence (5'→3')** |
| --- | --- |
| ***Schizochytrium* sp.** | |
| U6-1 | GACGTGAAGTCGTCCAACGTCATTCTTTTGCAGGAGCAATTCTACCACTATCTTCAGGGCTCGCCGTCTTTCTCTGCCGAGTGAACGCAGCTGGAGCTACGCTCGTCGTCCGGAAAACCGATTCGCAATCGTGTCCTTTCACCATGTTAACGCCGTATACTCGCATCAACGCTGTCACCTTTGCCTTGCTATTTCGATCCCTTTCTTTTTCGCTTTCCTGCCGCCCTCGCCGCCGTCGCACTGTACCAGCACTCTCTTACTGCTTATACTATATAAGTAAGAGTTACCCACTCTCGTTCG |
| U6-2 | GAAAGATCACTCGCAAGGTGAATGATCTCCCGCGGATTGCCCTCGCAGCTACTCACAACAGCGTGCTGCGCAACCTCCTGCAGCGTAGCCTCACGCTGCACCACATCTGAGTCCTGGTTGCTCCGAACAATGATCCTCCGCTTGAGGTGACGAACAAAGAGCAATGCGGCATCACCCGCATGCAGGGGCTGCAGCTGGTAGTTGACCACGTTCACATTGAGCGTGTGTCCCAAATGCAGCGGATTTTCCGAGGTAACGAGGATTCGTACGCCGGAAGTTTGGTCCAAAATTGAGTGCAAG |
| U6-3 | GTGTCTCAGATTGTTGACACAGGTTGCAGGTGCGGTTGGTGAGGAAGAGAGCCTCGAGTGCGTGATGTATGCAAAGTATCGGCTTGAAGACCAGTCTCACGGATAGTAGAAGGCGCCGCATCGTCAGCGAATCGTCAAAGCTGCGTGTTGAAGCTTGCGTAAGAGCCAGGCTGCGTTTCAGCGCTATATCAGCCGCGATCGGGGTGCGAGGAAGCGAGGAAGATTAAAAGTGAACAATATGGGCCAGCGTTGGCAACGCACGCGAGAGCACGCAAATGCAGTGCGATCATGGTCACAAAG |
| U6-4 | ACGACGCTGCTCACCTCTTCTGCAGGCAACGAAACCAACAAAGAGCTCGGCCCTAAACTTTTTATTTAAAAAAAAAGGAAAGGGTCAAACACACTAGCATGCTTTTCTCTTCAACGACACTCGCTTGATCCAAACGAAAACCATGGCACCAAGCAATGCGCCTGGGTCAGCCTTTGCAAGCCCATTTGAGGGTCAGCCGTCATTGTTGGCAGGCTGAGGCCGGGCCAAAGCAGTTCTACTTGGCCCAGCAACACCTCCTTCGCAACGCTAGCCTTGACTTACACAAGCAGCACGTTGCCA |
| U6-5 | CTGATTTACAGCGCTGCTCACTTCTTCCGCAGGCAACGAAATCAACAAAGAGCTCGGCCCTAAAATTTTTATTAAAAAAAAAGAGACACACACACTAGCATGCTTTTCTCTTCAACGACACTCGCTTGATCCAAAAGAAAACCATGGCACCAAGCAATGTGCCTGCGTCAGCCTTTGCAAGCCATTCACACTTCATCCGCCATTCCTTTCATGCCGCGAGCGGGCCAAAGCACATCAACTTAGCCCAGCAACACCTCCTTCGCAACGCTAGCCTTGACGTACACAAGCAGCACGTTGCCA |
| 5S rRNA-1 | TCGATCATACCACGTGGCCAACTCCTCATCTCATTCGCACTGAGCAGAAAAGTCACGTAGGGCCGGGTTAGTAGTGTGATAGGGGACTGCACGCGAAGTCCCGGTGTTGAATGTT |
| 5S rRNA-2 | TCGATCATACCACGTGGGCAACTGCTCATCTCATTCTGAGCAGAATAGTCACGTAGGGCCCGGTTAGTAGTGTGATAGGGGACTGCACGCGAAGTCCCGGTGTTGAATGTT |
| 5S rRNA-3 | TTGATCATACCACGTGGGCAACTCCTCATCTCATTCGCACTGAGCAGAAAGGTCACGTAGGGCCGGGTTAGTAGTGTGATACCGGACTGCACGCGAAGTCCCGGTGTTGAATGTA |
| 5S rRNA-7  (W2) | TCGATCATACCACGTGGGCAACTCCTCATCTCATTCGCACTGAGCAGAAAAGTCACGTAGGGCCGGGTTAATAGTGTGATAGGCCACTGCACGCGAAGTCCCGGTGTTGAATGTA |
| 5S rRNA-8  (W3) | TCGATCATACCACGTGGGCAACTCCTCATCTCATTCGCACTGAGCAGAAAAGTTACCTAGGGCCGGTTTAGTAGTGTGATAGGGTACTGCACGCGAAGTCCCGGTGTTGAATGTT |
| 5S rRNA-20 | TCGATCATACCACGTGGGCAACTCCTCATCTCATTCGCACTGAGCAGAAAAGTCACGTAGGTGCGGGTTAGTAGTGTGATACGGGACTGCACGCGAAGTCCGGGTGTTGGATGTA |
| tRNA-Trp(CCA)-1 | AAGGGAATAGCTCAATGGTAGAGCGTTGGTTTCCAAAACCAAAGGTTGTAGGTTCGAGCCCTATTTCCCTTG |
| tRNA-Glu(TTC)-1  (M1) | GCCCTTATAGTTTAATGGTTAGAACGTAGCTTTTTCGTAGCTTCAGTGAGAGTTCAATTCTCTCTAAGGGTA |
| tRNA-Leu(CAA)-2  (S3) | GCTCCTATGGCGGAATTGGTAGACGCGGTAGATTCAAAATCTATTTTTTTGCTGGTTCAAATCCTGCTAGGAGTA |
| tRNA-Gln(TTG)-1 | TGGGGTATAGCTTAATGGTAAAGCTTTAGTTTTTGGTACTAAATATTATAGGTTCGAATCCTGTTACTCCAA |
| tRNA-Arg(CCT)-1  (S1) | GTCCCTGTAGAATAGTGGTTAATTCGTCTCCCTCCTAAGGAGAAGGGCCTGGGTTCGACTCCCAGCAGGGACT |
| tRNA-Thr(TGT)-1 | GCACCCGTAGTACAGTGGTAGTTACGTCGCACTTGTAATGCGAAGGTCACGAGTTCAATTCTCGTCGGGTGCA |
| tRNA-Tyr(GTA)-1  (W1) | CTCGAGATAGCTCAGTTGGTAGAGCGTTGGATTGTAGCGTTTAACCAGAAGCGATTCTGGTTGTCTGATTTCCACTGGTCTTCGGTTCGACTCCGGATCTCGAGA |
| tRNA-Ala(AGC)-3  (M3) | GGGCACGTAGATCAGTGGTAGATCGCCTACTTAGCATGCAGGAGGGCATGGGTTCAATTCCCATCGTGTCGA |
| tRNA-Gly(TCC)-1  (S2) | GCGGATGTAGTGTAATGGTTAACACCTTAGCCTTCCAAGCTAATATTGAGGGTTCGAATCCCTTCATCCGTT |
| tRNA-Gly(GCC)-1  (M2) | GCGCTGGTGGTTTAGTGGTAAAATAGCTGTTTGCCATGCGGCTGCCCCGGGTTCGATTCCCGGCCAGCGCT |
| tRNA-Met(CAT)-1 | GGCGGTATAGCTCAGTTGGTTAGAGCATGGGAATCATAATCCTGGAGTCGCAAGTTCAAATCTTGCTACCGCTA |
| hU6 | GAGGGCCTATTTCCCATGATTCCTTCATATTTGCATATACGATACAAGGCTGTTAGAGAGATAATTGGAATTAATTTGACTGTAAACACAAAGATATTAGTACAAAATACGTGACGTAGAAAGTAATAATTTCTTGGGTAGTTTGCAGTTTTAAAATTATGTTTTAAAATGGACTATCATATGCTTACCGTAACTTGAAAGTATTTCGATTTCTTGGCTTTATATATCTTGTGGAAAGGACGAAACACCG |
| ***Yarrowia lipolytica*** | |
| SCR1′-tRNA_Gly_ | CCCCAGTTGCAAAAGTTGACACAACTCTAGATCTGCTTCCAAATATAGAATCATAACAAGGGTTAGGGTGTGATTATATAATATTGGTCTTAATTGATGTGCTAGGGCTTTAAAAGTTGGTTAAAATAACGCTCTAATGCCTTTTTAATATATTGTCTTTTTCAAAATCTCAAATCGGACACTTCTTCGTGTATGAGACTCCATTTTTTGGCTCCGTCACGTGATATGTATTATCAGCTATAGTGGTGTAAACAAAGTTTTTTACTAGCTGTAATGGCATTTTGTCGGAGTGGTAAATCGCCTTCTTGTTGTGCGTTCGAGTTCTGGACTCTGCACTGGGCTACTTTGAAAAATACCTCTAATGCGCCGATGGTTTAGTGGTAAAATCCATCGTTGCCATCGATGGGCCCCCGGTTCGATTCCGGGTCGGCGCAGGTTGACGT |
| RPR1′–tRNA_Gly_ | TTCATTACAGCTATATACCTAGTAAGCCGGGTTATTGGCGTTCAATAAATCATACACTTCTGAATCTTTGATTACAGTCATTCCTGACGACTGTCCGATGAAACGGCCTAAAAAAGTTAAAATGTCGGGAAATTTAGCTCTGGCCTAACGGTCAGACTGCAGTTACCCGGCCTCTCCCTCTGGCCTCGCGTACCTGGCTACACAAGACTGCGCTCTGCAGTTCGAATCCAGGTGGGAAATTCGGTGTGAAAAATACCTCTAATGCGCCGATGGTTTAGTGGTAAAATCCATCGTTGCCATCGATGGGCCCCCGGTTCGATTCCGGGTCGGCGCAGGTTGACGT |
| SNR52′–tRNA_Gly_ | GACCCCGTCTTCAATTACACTTCCCAACTGGGAACACCCCTCTTTATCGACCCATTTTAGGTAATTTACCCTAGCCCATTGTCTCCATAAGGAATATTACCCTAACCCACAGTCCAGGGTGCCCAGGTCCTTCTTTGGCCAAATTTTAACTTCGGTCCTATGGCACAGCGGTAGCGCGTGAGATTGCAAATCTTAAGGTCCCGAGTTCGAATCTCGGTGGGACCTAGTTGAAAAATACCTCTAATGCGCCGATGGTTTAGTGGTAAAATCCATCGTTGCCATCGATGGGCCCCCGGTTCGATTCCGGGTCGGCGCAGGTTGACGT |
| tRNA_Gly_ | TGAAAAATACCTCTAATGCGCCGATGGTTTAGTGGTAAAATCCATCGTTGCCATCGATGGGCCCCCGGTTCGATTCCGGGTCGGCGCAGGTTGACGT |
| SNR52 | GACCCCGTCTTCAATTACACTTCCCAACTGGGAACACCCCTCTTTATCGACCCATTTTAGGTAATTTACCCTAGCCCATTGTCTCCATAAGGAATATTACCCTAACCCACAGTCCAGGGTGCCCAGGTCCTTCTTTGGCCAAATTTTAACTTCGGTCCTATGGCACAGCGGTAGCGCGTGAGATTGCAAATCTTAAGGTCCCGAGTTCGAATCTCGGTGGGACCTAGTTATTTTTGATAGATAATTTCGTGATGATTAGAAACTTAACGCAAAATAATGACGT |
| 5S rRNA | GGTTACGGCCATATCCTGGTGAAAATACGGCTTCCCGTCCGATCAGCCATAGTCAAGCACCAGAGAGCCTAGTTAGTATTGTAGTGGGAGACCATACGAGAATCCTGGGTGCTGTAATC |

**Supplementary Table 4. Coding sequences of genes overexpressed in this study**

| **Gene** | **Sequence (5'→3')** |
| --- | --- |
| ***Schizochytrium* sp.** | |
| eGFP | ATGGTGAGCAAGGGCGAGGAGCTGTTCACCGGGGTGGTGCCCATCCTGGTCGAGCTGGACGGCGACGTAAACGGCCACAAGTTCAGCGTGTCCGGCGAGGGCGAGGGCGATGCCACCTACGGCAAGCTGACCCTGAAGTTCATCTGCACCACCGGCAAGCTGCCCGTGCCCTGGCCCACCCTCGTGACCACCCTGACCTACGGCGTGCAGTGCTTCAGCCGCTACCCCGACCACATGAAGCAGCACGACTTCTTCAAGTCCGCCATGCCCGAAGGCTACGTCCAGGAGCGCACCATCTTCTTCAAGGACGACGGCAACTACAAGACCCGCGCCGAGGTGAAGTTCGAGGGCGACACCCTGGTGAACCGCATCGAGCTGAAGGGCATCGACTTCAAGGAGGACGGCAACATCCTGGGGCACAAGCTGGAGTACAACTACAACAGCCACAACGTCTATATCATGGCCGACAAGCAGAAGAACGGCATCAAGGTGAACTTCAAGATCCGCCACAACATCGAGGACGGCAGCGTGCAGCTCGCCGACCACTACCAGCAGAACACCCCCATCGGCGACGGCCCCGTGCTGCTGCCCGACAACCACTACCTGAGCACCCAGTCCGCCCTGAGCAAAGACCCCAACGAGAAGCGCGATCACATGGTCCTGCTGGAGTTCGTGACCGCCGCCGGGATCACTCTCGGCATGGACGAGCTGTACAAGTAA |
| RdRP | ATGGCCGTCTCGCTCAACGACTTTATTTCGGTCAAGCTCAAGCGCTACTCGCGCGAGTCGCCCTGGGAGCGCCTCCGCGTCCCCTACCGCAACAAGAAGCAGAAGAAGTGGGCCTCGGTCCACAACAACGAGGCCCAGCTCCACTCGGCCAACAAGCGCAACGACAACTGCCTCATTCAGCGCTCGTCGACGTGGCGCCTCGGCGACATGATTACGCTCGTCATTAAGGACATTCCCGTCACGTGGCTCTCGAACGAGGGCGGCAAGCTCTACAACCTCTGGGAGCCCCTCCACGACTACGGCACGATTGAGTTTATGAAGATTAACGAGCCCCTCAACGGCCAGACGTCGACGACGGCCATTGTCCAGTTTGCCCCCCCCCCCAAGGTCCCCTTTTGGGAGCCCAACGGCAAGATTAACGTCAAGGGCGTCGACCTCGCCGTCCAGATTGACATTACGGCCCACCGCTCGCACATTTCGCGCCAGGTCTTTTCGAAGAACTCGTTTCGCTCGGACCAGCTCGTCAAGATTCCCCTCTCGTCGTTTAAGCTCGGCCAGGTCTACGACGAGCGCATTGTCCCCCTCTTTGGCGTCGACTGCGGCATTACGGTCACGGAGTCGAACCTCCTCGTCTACTTTAACTTTAAGAAGCTCTGCGTCCTCTTTGACGCCTCGTTTGACAAGCAGATTGAGACGTTTCGCCTCGACTTTGACTTTCACTCGATTATTGGCGACGTCGGCACGGACTACTACGACGACCACATTTCGCTCGTCTTTCGCTTTCGCTTTTCGCCCCTCATTTTTCGCAAGTCGAAGAACGCCACGGAGTCGCGCGTCCAGACGTTTTGGACGGCCTCGCACCTCTGGCGCCGCCACTACGACATTCTCCCCTTTAACGTCTCGCCCACGACGGCCTCGCCCATTGAGCTCCTCAACTGCCACAACGCCCCCATTGGCCGCTGCAACGTCCTCGTCCTCTCGTTTTCGATTCGCGACGAGTCGGACAAGGACGACATTGCCTTTCTCCTCCACAACCTCGAGAAGTTTAACCTCAAGTCGCAGCTCGACAAGGTCGTCTTTCACCTCGTCCCCGACTACAAGCACCGCTGCTCGCTCATTAACGACAAGGAGATTGAGGAGGAGATTGCCTACCTCCTCCAGGCCTGCCTCTCGAAGAACCTCCTCTCGGAGATTGACCTCCCCATTATTCTCGCCAACCTCAAGAAGCTCTCGAAGGAGCGCGCCAAGAAGTTTCTCCGCCTCATTCTCACGTCGAAGACGGCCCTCATTAACCCCTCGGAGCTCGACTTTACGAAGTCGTTTGTCTTTTACGACCTCTCGTCGGCCTCGTCGATTCACATTAAGAAGCTCTACGTCACGCCCACGACGCTCCGCATTGTCGAGGACTCGCTCGAGGCCGGCAACCGCGTCATTCGCAACTTTAAGGACTTTGCCAACCGCTTTATGCGCGTCCAGATTACGGACGAGTACTACAAGCAGAAGATTCGCGGCGGCTCGGACGGCTTTCGCAACGAGAAGCTCTACTCGCGCATTCAGCAGCTCCTCACGTACGGCATTAAGGTCGGCAACCAGATTTACGAGTTTCTCGCCTTTGGCAACTCGCAGCTCCGCGAGCACGGCGCCTACTTTTTTGCCTCGGGCTCGGACCTCAACGCCAAGCAGATTCGCGAGTGGATGGGCGACTTTTCGGAGATTAACTCGGTCTCGAAGTACGCCGCCCGCATGGGCCAGTGCTTTTCGACGACGAAGGAGATTAACCGCTTTTGCGTCGACATTTCGCTCCAGGACGACATTGTCCGCAACAACCACTGCTTTACGGACGGCGTCGGCATGGCCTCGCTCTCGGTCATTCGCCGCCTCTCGCTCGAGGTCAAGAACCACGACATGTTTCCCTCGGCCTTTCAGTTTCGCATGGGCGGCTACAAGGGCGTCCTCTCGCTCGCCCCCCCCACGAAGCTCGAGTACCACCAGGGCAACCTCGTCTTTCCCCGCCGCTCGCAGGACAAGTTTAAGTCGTTTCACTCGACGCTCGAGGTCATTAAGATTTCGCGCTTTTCGAACGCCCACCTCAACATGCAGCTCATTACGCTCCTCGAGGGCCTCGGCGTCGAGAAGACGGTCTTTCTCGAGCTCACGCGCTCGCAGCTCTCGAAGATGAACGAGTCGATTAACTCGAAGCAGAAGTCGATTCTCATGCTCCGCGACAACGTCGACGAGTACCACTCGACGCTCATTATTGCCGACTTTATTCAGGCCGGCTTTCTCGAGCGCGACGACGCCTTTACGGAGAACCTCCTCAACCTCTACTACGAGTGGGTCCTCCGCCTCATTAAGGAGAAGCAGAAGGTCTCGGTCCCCAAGGGCGCCTACCTCCTCGGCGTCGCCGACGAGACGGGCACGCTCAAGGGCCACTACGACGACGCCGTCCTCTCGGTCCCCGAGATTTTTATTCAGATTACGGACACGTCGACGTCGTTTGGCTCGTACTCGACGGGCAAGCTCAAGACGCGCGTCATTGTCGGCCTCTGCATTGTCGCCCGCAACCCCTCGCTCCACCCCGGCGACGTCCGCGTCTGCAAGGCCGTCCGCTGCGACGAGCTCATGCACCTCAAGAACGTCATTGTCTTTCCCACGACGGGCGACCGCTCGATTCCCGCCATGTGCTCGGGCGGCGACCTCGACGGCGACGAGTACACGGTCATTTGGGACCAGCGCCTCCTCCCCAAGATTGTCAACTACCCCCCCCTCCTCGAGTCGTCGCCCAAGAAGTCGATTGACTTTCTCGAGGGCAAGCCCCTCATTGACTCGGTCAAGGAGTTTTTTGTCAACTACATTAAGTACGACTCGCTCGGCCTCATTTCGAACGCCTGGAAGGCCTGGGCCCACGACCACGACAACAACCCCGAGGGCATTTTTGGCAACGTCTGCCTCGAGCTCGCCGAGATGCACTCGAAGGCCGTCGACTTTGCCAAGTCGGGCGTCGCCTGCAAGATGCAGGCCAAGTACCACCCCAAGCGCTACCCCGACTTTATGCAGAAGACGAAGACGCGCTCGTTTCGCTCGGAGACGGCCGTCGGCAAGATTTTTCGCTACGCCGCCCGCTTTCAGCGCGAGTCGGGCCGCCCCGCCACGTACAACCCCATTATGAACACGGTCTACGACCCCTGCATGAAGCTCCCCCGCTTTAAGACGGAGTACCTCAACGTCGCCGAGGAGGTCAAGAAGCACTACGACAACGACCTCCGCTCGATTATGGCCCGCTTTGACATTTCGACGGAGTACGAGGTCTACACGGCCTTTATTCTCTTTAAGGACGACCTCGCCAAGACGGTCAACGAGTACGGCCTCCGCGAGGAGGTCTCGTTTCAGTTTGACCTCCTCAAGAAGAAGTACACGCAGGAGTACCTCGAGAAGTGCGCCCTCTCGAACCAGTCGGCCTTTGACTCGTCGGAGTACGAGGAGCGCATTAACTCGGCCGTCGCCGCCACGTACGACGTCACGTACGACCAGCGCGTCAAGTCGGTCGGCAACGGCACGACGGAGGTCCTCATTTCGTTTCCCTACCTCTTTTCGTCGCGCCTCTGCCAGCTCTCGCGCAAGGCCATGCTCACGGCCAACAACTTTTAG |
| SsΔ9D | ATGACCGAGCAATTGGACACCGCGGACATCACCGCCCTCAATGCTTTGGCTGCCGGTACCAACAAGAAAACTCCCAGAATCGTGGCTGCTGGGTTGGGAGGCAGATTGATGGGTACTGCCCAAATGGTCAAGGTGACTGCCGAACAGATCACTGAGGACTCCTTGGAGTCCTTGGCCGACAAGGACGCCCGTGAAAAGGCAATGTACGCCAAGACTAAACACATCTCCGAATTGCCATGGACGTTTGGAAACTTCTTTGAGAAGATCAACTGGTTGAACATGATTTTAGTGGTAGCCTTGCCAGCCTTCGGTACCTACCAGGCTATCTTCTACCCTCCACCTTTGAAGACCTTGGTTCTTGCCTTCGTGCTCTACCTCTCCTGTGGTCTTTCCATCACTGCTGGCTACCATCGTTTATTTTCCCACAGATCCTATGACGCCGTCACTCCAATCAGACTCTTCTTTGCCTTCTTTGGTGCTGGTGCCATCGAGGGTTCCATCAAATGGTGGGGCCACTCGCACAGAATCCACCACAGATACACCGATACCGACAGAGACCCTTATGATGCCAGACAAGGTTTCTGGTACTCCCACATGGGATGGATGTTGACCAAGGCCAACCCAAAGAACAGAGCCAGAGCCGACATCTCAGACTTGTCCACCGACTGGGTCGTCACTTTCCAGCACCGTCACTACTTGTTGATCATGCTTGTATCGGCCTTTGTAGTTCCTACCTTGACTGCTGGTCTCTTGTGGGGCGATTACTGGGGTGGTTTCATCTACGGAGGCATTTTAAAGTCTTTCTTCATCCAACAAGTCACTTTCTGTATCAACTCTTTGGCCCACTGGATCGGTGTTCAGCCATTTGACGACAGAAGAACTCCAAGAGATCACGCATTGACTGCTGTGTTCACTTTCGGTGAAGGTTACCACAACTTCCACCACGAGTTCCCATCTGACTACAGAAACGCTCTTAAGTGGTACCAATACGACCCTACCAAGATCGCTATCTGGATCTTGTCCAAGCTTGGTCTTGCCTGGAACTTGAAGAAGTTCTCGCAAAACGCCATCGAGCAAGGTTTGGTTCAACAACAACAAAAGAAGTTGGACAGAATGAAGTCCAAGCTTAACTGGGGTCTTTCTGTCGACGAATTACCAGTGTGGGACAGAGAAGAGTTCAACGCCAAGGCAAAGACCGATGGTCTTATCATCATCTCTGGTATCATCCACAATGTCAAGAACTTCATCAAGGAACACCCTGGTGGACAAGCATTGGTCAGAGCCTCGTTGGGTAAGGATGCCACTAAAGCTTTCAATGGCGCTGTGTATGCCCATTCTAACGCTGCCCACAACTTGTTGGCTTCGATGAGAGTGGCCGTGATAAGAGACGGAAACATCAACGCCAACACTTTTGATCTTCAACAACAGTTCTTGGACAAGGAGAAGCTTAACTAG |
| GGPPS | ATGGCCTTTTCCACGAGACCGACGCTCATGGCCAAGCTCGCCGCGGGGCCCGCGGCGCCCGCCGGCAGGGGCGGGCCCGGCGCCAAGCAGGCCGAAATCGGCGTCGACGCGGCGGAGAGGAGCCTCGACCTCGACGTCGAAGAGGCAGGGCCCGAGGAGGAGATGAAGCAGGCCGCGGTACACGAGATGAGGGCGCATGACGTGGGCCCCGGGGCGCCCGCCGCCTCCGCCTCGGTCGCCGCTCTTGGGCAGCATCAGGTCGCCGAGGAATGTCTCGAGCCCTGGCGGTACATCTGCAAGACCCCGGGCAAGAACGTGCGGTCCAAGATGATTGACTGTTTCCAAGAGTGGCTTCGCATCCCTGAAGAGAAGCTACCGATCATCAAGCAGATCGTCGGATCGCTACACAACGCGTCACTGCTCATTGACGACATCGAAGACGACTCAAAGCTGCGTCGCGGGCAACCCGTAGCCCACTTGATCTATGGCATCCCGCAGACGCTTAATTGCGCGAACTATGTATACTTTATGGCCATGGAAAAGTGCCTGGCACTGCAAAACCCGTTCGCCCTTCAAGTCTTCACGCTCGAGCTCCTAAACCTGCACCGGGGCCAGGGAAGGGAACTCTTATGGCGCGATACAGGCAAGTGTCCTAGCGAAGACGACTATCTTGGCATGATCATGGATAAGACGGGTGGGCTCTTCCGTCTTGGCGTGGGGCTTATGCAGAGCTTTTCCGAATCGCGCACGGACTTCTCCAAGCTCGTCAATCTGCTTTCCATCTACTTTCAAGTGCGCGATGACCTCATCAACCTCGCTAGCAGCTCGTACCACCGCAATAAGAGCTTTTGCGAGGATCTCACAGAAGGAAAATTCTCTTTTCCCATTATTCACGGGATCAAGAACTCGGAGCCAGGAGATAATTACCTCTTGTCTATCCTCAAGCAGCGCACCGAAAATGTTGATCTCAAGCGAAGTTTTGTCGATTTTCTCCGTACGGAAACTCGATCCTTCGCTTATACCAAGCAATATTTGGATCGTATTTTCCAGGACATTCAACAAGAGATTAGCGCTGTAGGCGGTTCCCAACATCTCATGACGCTAATGGAGAAACTTCGTGAAGAAGTTGACGACTGCGATATTTGA |
| PSY | ATGGCGCGCATGGGCGTCAGCATGGGCAGGGGCATGGATGTTGTGGGCTTGAGTCTCGGCGGCCTGCGGCTGCGCGCCGGAGGACGTAGCGCCTCGGCCACAGCCTTGCGAAGACCCAGAAGCTGGCAAGAGGCCGGAGCATGGGCCAGGGCCGCCTCCTCGAGCGCTGGACCTGGATCGGACGCCGGGCACGCAGCACCGGCCGGGGCTGCCGATGCTGCGCAAGTCTGCGCTGCCGAGGTCCGAAAGGGCGATCGACACGCGTATCTGGCCGGCGCCCTCATGCCCTCGCATGTGCGTAGGCAATTCTATGCTGTGCGCTATTTCAACCTCGAGCTCGCAAGATTGCGCGATGTCACAGGCGCAAATGAAGCTGCTGTCTCGCTTCGGTGCCAATGGTGGCGCGAACTTCTTGGAGAGGCCTTTGCAGCTGCCAAGGAGGACCCGAGCTTGCATACCGCGACGATCGCACAGTCTCAATATGGTCCGCGCGGTCATCCCGTCTTTTACGAGCTTACCCATGCCATTCGCCAGAGCAAGCTGACCCGCCGCTGGCTTGACCGTATGATTGACGCTCGCCAGGACGATGCTATTACAGAGCAGCCCGCCTCGCTTGCAGACATGGAGAAGTACGCAGAGTCTACCGCCTCGAGCATGCTTTACCTTTCGCTTGAGATGTTTGGCGTTCGTGAGACGAATGCCGATCATGCCGCGAGTCACGCCGGCAAAGCCATCGGTCTCGCGAACACGCTTAATGGTCTCCCGTATCTTCTCGCCAGCGGGCAGATCCCGCTTCCGCAAGATCTTATGACCAAGCATGGTCTGCGCACTCGTAGCTTTCAGCGCTTGGCGATGATGGCAGGTGTTGGCATGGGTCAGCAGGACAAGCTCAACGCTGGCCAGGGTCCTCAGTCGATCGAGGAAGATGAGGAGCTCGTGCGCGCTTATCAAGCGCTCACAGAGACTGTGTATGATGTGGCAGCTACTGCGCACGGCCACCTCGAGCACGCAAGAGAGATGCGCGAAGCCATTCCGCCCGCTGCCTTGCCCGCTTTCCAGGTCACTGTCCCTACCTCCATCTTTCTCGAGCGTCTCCAGTCCACTGCAGAATTTGACATTTTCAATGCAAACTTGCGCAATGATCCCTTTGGTCCTCTCCGCGTCCAGTATCACATCTTCAAGAATAACTTCCTAAAGACCTTCTAG |
| LCYB | ATGGCGCGCAGGGCGTCGCGCCTCGGCGCCGCCGTCGTCGTCGTCCTCGTCGTCGTCGCCTCCGCCTGCTGCTGGCAAGCCGCTGCGGACGTCGTGGACGCGCAGGGCGCAAGAGGCCCGGGGCAAGAGAGCGACGGCGGCCGCGCGAAGAAGCGCATCGCCGTGCTCGGGGCCGGGTACGCAGGCCTGTCCGCAGCCTGCGAACTGAGCAGACTGGGACACGAGGTCGTGGTTCTCGAGAAGAACGCCTACGTGGGAGGCCGTGCCCACCAGTTCGAGGTCGAGGCCGACAATGGGCAGACCTTCAAGTTCGACGCCGGGCCCAGCTGGTACTGGATGCCCGAGGTCTTTGACCGCTTCTTTGCGCGGTATGGGCGAACCGTCCAGGAGTTCTACCAGCTCGAGCGCCTCGACCCGGCATATCGCATCATTCGCAATGACCACAACGGCGAGGGTACCGTCGATGTGCCCGGCGCTTCGAGCGAGGCCTTCATGTCTTGGGCACGCCAATTGAACGGCGATGCCCGACTCGTCGACCGTCTCATGGACGAGGCCAAGGCAAAGTACGAGGAGGGCGTCTTCAAGTGGATTTGGCATCCCATGGTCTCGTGGTGGGAAATGATCGATCTCAATCTCGCGCGCGCTGCCTTGCAGTATGACATGTTCAACAGCTTTGTCGCTCACCTGCAAAAGTACATTTCAAGCGATACCCTGCTCATGATTCTCAAGTGGCCCGTCATCTTTCTCGGGGCCTCGCCTAATGGCGCCCCTGCGTTGTATTCCATGATGACCTATGGCGGTCACGCGCTCGGCACCTTTTATCCAACTGGAGGCCTCGCGCGGCCCGTCGTTGCCATCGCCGAGCTTGCCAGAGACCTCGGCGTCGACATTCAGCTCGATGCCGAGGTCACCTCGTTTCGCTTTGACGAGAGCGGCCGTGGTGTTCAAGCTGTTTGCACTCGCAACGATCGCTGTGAGGCTGTCGATGGGGTCGTGGCTGCCGCCGATTACCACCACGTTGAGCAGACCCTTCTGCCCCCGGAACTTCGTCGCTACGAGCAGGGTTTTTGGGATGCCCAAGTCATGTCGCCGTCCTGCGTCCTCTTCTACCTCGGCTTCGATCACCGCATCCAAGGGCTCACCCATCATACGTTCTTCTTTGACCGAGACCTCGACGCTCATCTTCACGCGGCCTTTGACACGCACACTTGGGCCGAGGAACCCGTCTTTTACGTGTCAGCCACCTCGAAAACGGACCCAAGCGTAGTTTCTGGTCAGGGCGAGGCGCTCTTTGTGCTCGTTCCCATCTCCTACCAGCTCAACGGCACAGACAACGCTGCGCGTCGGGAGCAAATCCTACACACCGTGCTCACACGCATGGAAGAGAACTTGAAGCAGCCCCTCCGCGAGTGGCTCGTCTACCAAAAGTCCTACGGGACAACGGATTTTGAGCGCGACTTTCACTCCTTTCGTGGCAATGCTTTTGGCCACGCCAACACGCTTTCGCAGTCGCTCGTGCTCAAACCCTCCATGGACTCTTTACTCAATAATCTCGTCTTTGCTGGCCATTTGACGAATCCCGGCCCAGGCGTGCCGCCGTCCATCGTCTCGGGCACTGTTTCGGCCAACCTGTTGCATGACAAAATCCAAGTGACAGCAAATCACCATGCACTGGGGTTCACGCTCCTCGGAGCTTTCCTTGGAGCCTTGTTACTCGGTATTCTGGCTCTCTCGGTATTCTCAACTCGCTTCGTGTCGTATGTGGAGTGCATCAGACTCTTGTATGTGCACGGGCGGACTTACTTTGCCGCCGCCACGCTCATGAAGCCCATGGCCTTTCTCGACACGGCGGCCATGTACGGGCTTTTTCGCGTTGCCGACGACTACGTCGACAATGTTGGCGACGCCGGCGAGCGGCAGCGGAACCTCGACGCCTTCATGGCGGACTTTTGGCGATGCTGGGAATCCGGCCGAGGCGACTACGCGCGCCATCCGACGCTCCCTGCCATCATCGAGTCGGCGCACCGTCGTGCATACCCGCGGGAACTCTTTGAGCGTTTCTTCCGCTCCATGCGGATGGACGCCAAACGAAAGGTCGTCTGCCTCACCATGGATGATACGATGGAGTACATGGAAGGCAGCGCGGCTGTCATTGGCGAGTTCATGCTACCTATTCTCATGCCCGACAGAGACTCTTTGGCTTTCAAGCAAGCCGTACCGCACGCGCGCAATCTTGGACTCGCTTTCCAAATCACCAACATGCTTCGGGATATTGGCGAGGATAATCGCTTGGGTCGCCAGTACATTCCTGTCGACGCCTGCAAGCGCCATGGTCTAAACGGCAAGCTCACGTCTCATGAACAGCCTGGCTTTCGCGAGCTCATGGAGGAAATGTTCGCTTTCACCGACAATCTCTATGCTAGTGCTGACCTTGGCATCGACATGTTGCCTGAGCAGGTGCGCGACGTCATTCGTGTGGCGCGTCTTGCGTATCACCGCATCCACGACAAGATCCGCGCAGCGAATTACGACATTTTCACCGCTCGACGTCGAGTTCCCCTTGGAGAAAAGTTAACGATTCTCGTCGATACCGTACCGCGACTCAAGCTCGCCCGCATTGCGCTCACCGAGCTCATTTGTGCTACACTCTATGGTCTTTCGCGCCCGCATATTGCTTTCGTTTGGATTGGCGCCGTATGGGCGAGCTGGCTCGAGTGGCCGGGATGCTCGTACCTGCGCTTTCACGGGCTCTTCATACTACCGCCGCTCCTCATGCTCGCCCGTTTGGCGCACCAACGCGCTGTTGCCGACAAGCAGGTCCCCTTCTTGCGCCGCGCTGGTTTCTGGACTGTGGCACTTTGCGTCGTTGCAACACTTTACACCACACCATGGGACAATTTTCTCGTGTATCGCCGCGTCTGGGGATACCCGCCGGAGCGCATTCTCTTTGTCATTGGGTATGTGCCCATTGAAGAGTACATGTTCTTCACGCTCGAAACCATGTTGGTCGCGGCGGTCTGGCTACAGGTTTTTCAGCCCACGACGTTGCAGGCCGAGGTAGGCCCACGTGGAAAGGGGGGCATGCTCGTTCTCGCGAGTCTTGGACTCGTCTGGGTTGCCGGCCTTTCGTGTTTGGCCTCGGAGCAAAGCTTATACATTGGTCTCATTCTCAGCTGGTCTATGCCCGTCCTCATTCTGCAATGGAGTCTCGGTGCACATGTGCTCACTACGCATGCAAAGCCGGTCCTGACGACGATCGTGTCGGCCACAGCGTACCTTTGCGTGGCCGACGAATGGGCGATTCGTCACGGCATCTGGCGCATCAATCCTGCAAATCTTGTGTTGCCCATGGGCAAACATGCACTTCCCCTCGAGGAAGCCCTCTTCTTCTTGGTGACCTCGATCATGTGTACGTGGGGACTCACGCTGGCCATGGTTCTCTGGGGCAAGCCCATCGGCTTGGCAGTTGGTATGGGCACTTGGGCGAGACCGCCTCGTCCGGGCCGGACGCAACTCATTACCTGTGGAGCCGTGCTTGTGTTGAGCATTTCTCATCCGGCGCTGTTTACGATGGTTCCTGCACTTGTGGTCACAATCATGCGCTTTGGGTTTTGGGCGTGCACACTCATGGCCGGCGTGCATTTGCCTGCTCGTGGGCGTATTCTCTTTGTTGCTGCAGTCGTGGCAATTTCTTGTGCGCCTACTGCTCTGGCGCCACTTTTGGCGGGGGCTGTTCTTGTGGTTTCGCTCGGCGGCTGGCATACGCGAGGTCGCGACGACACGCTCCCGCTGTACAAGAATGCTTGA |
| ***Yarrowia lipolytica*** | |
| yl16E | ATGCTCTCGTCAATCTCGCCCGACCTATACTCGTCCTTCTCGTTCAAAAACTCGCTCGCCGAGGCCATGCCCTCCGTGCCACACGAACTCATCAACTCAAAAACACTCTCATGGATGTACAATGCCTCTCTGGACATTCGGGTTCCTCTGACTATCGGAACCATCTACGCCGTCTCCGTGCACCTGACCAACTCATCTGAACGAATCAAGAAACGCCAGCCCATTGCCTTTGCCAAGACCGCACTCTTCAAGTGGCTCTGTGTCCTCCACAATGCAGGTCTGTGTCTCTACTCAGCATGGACCTTTGTCGGTATCCTCAACGCCGTCAAACACGCCTACCAAATCACAGGAGACAGCTCCGCCCCCTTCTCCTTCAACACCCTCTGGGGATCGTTTTGTTCACGTGACTCCCTCTGGGTCACCGGCCTCAACTACTACGGATACTGGTTCTATCTGTCCAAATTCTACGAAGTGGTGGACACCATGATCATCCTCGCAAAGGGAAAACCGTCCTCAATGCTCCAGACATACCACCACACCGGCGCCATGTTCTCCATGTGGGCCGGCATCCGATTCGCCTCTCCCCCCATCTGGATCTTTGTGGTTTTCAACTCCCTCATCCACACAATCATGTACTTTTACTACACCCTCACCACCCTCAAGATCAAGGTTCCCAAGATCCTCAAGGCATCTCTGACCACCGCCCAGATCACCCAGATTGTCGGAGGTGGCATCCTGGCTGCCTCCCACGCCTTTATTTATTACAAGGACCACCAGACTGAGACAGTCTGTTCTTGTCTCACTACCCAGGGTCAGTTTTTCGCTCTCGCCGTCAATGTCATCTATCTGAGTCCTCTGGCCTATCTCTTTATTGCCTTCTGGATTCGATCTTACTTGAAGGCCAAGTCCAACTAG |
| ylΔ9D | ATGGTGAAAAACGTGGACCAAGTGGATCTCTCGCAGGTCGACACCATTGCCTCCGGCCGAGATGTCAACTACAAGGTCAAGTACACCTCCGGCGTTAAGATGAGCCAGGGCGCCTACGACGACAAGGGCCGCCACATTTCCGAGCAGCCCTTCACCTGGGCCAACTGGCACCAGCACATCAACTGGCTCAACTTCATTCTGGTGATTGCGCTGCCTCTGTCGTCCTTTGCTGCCGCTCCCTTCGTCTCCTTCAACTGGAAGACCGCCGCGTTTGCTGTCGGCTATTACATGTGCACCGGTCTCGGTATCACCGCCGGCTACCACCGAATGTGGGCCCATCGAGCCTACAAGGCCGCTCTGCCCGTTCGAATCATCCTTGCTCTGTTTGGAGGAGGAGCTGTCGAGGGCTCCATCCGATGGTGGGCCTCGTCTCACCGAGTCCACCACCGATGGACCGACTCCAACAAGGACCCTTACGACGCCCGAAAGGGATTCTGGTTCTCCCACTTTGGCTGGATGCTGCTTGTGCCCAACCCCAAGAACAAGGGCCGAACTGACATTTCTGACCTCAACAACGACTGGGTTGTCCGACTCCAGCACAAGTACTACGTTTACGTTCTCGTCTTCATGGCCATTGTTCTGCCCACCCTCGTCTGTGGCTTTGGCTGGGGCGACTGGAAGGGAGGTCTTGTCTACGCCGGTATCATGCGATACACCTTTGTGCAGCAGGTGACTTTCTGTGTCAACTCCCTTGCCCACTGGATTGGAGAGCAGCCCTTCGACGACCGACGAACTCCCCGAGACCACGCTCTTACCGCCCTGGTCACCTTTGGAGAGGGCTACCACAACTTCCACCACGAGTTCCCCTCGGACTACCGAAACGCCCTCATCTGGTACCAGTACGACCCCACCAAGTGGCTCATCTGGACCCTCAAGCAGGTTGGTCTCGCCTGGGACCTCCAGACCTTCTCCCAGAACGCCATCGAGCAGGGTCTCGTGCAGCAGCGACAGAAGAAGCTGGACAAGTGGCGAAACAACCTCAACTGGGGTATCCCCATTGAGCAGCTGCCTGTCATTGAGTTTGAGGAGTTCCAAGAGCAGGCCAAGACCCGAGATCTGGTTCTCATTTCTGGCATTGTCCACGACGTGTCTGCCTTTGTCGAGCACCACCCTGGTGGAAAGGCCCTCATTATGAGCGCCGTCGGCAAGGACGGTACCGCTGTCTTCAACGGAGGTGTCTACCGACACTCCAACGCTGGCCACAACCTGCTTGCCACCATGCGAGTTTCGGTCATTCGAGGCGGCATGGAGGTTGAGGTGTGGAAGACTGCCCAGAACGAAAAGAAGGACCAGAACATTGTCTCCGATGAGAGTGGAAACCGAATCCACCGAGCTGGTCTCCAGGCCACCCGGGTCGAGAACCCCGGTATGTCTGGCATGGCTGCTTAG |
| ylΔ12D | ATGGCCTCAACGAGTGCACTGCCCAAGCAGAACCCTGCTTTGCGTCGCACGGTTACCTCCACCACCGTGACCGATTCTGAATCTGCAGCTGTGTCACCCTCTGATTCTCCCCGACACTCTGCCTCCTCGACTTCTCTGTCATCCATGTCTGAGGTCGATATTGCCAAGCCGAAGTCCGAGTACGGCGTCATGCTTGACACCTATGGGAACCAGTTTGAAGTCCCCGACTTCACAATCAAGGACATCTACAACGCCATTCCCAAGCACTGCTTCAAGCGTTCTGCTCTTAAAGGATATGGATACATTCTCAGAGATATCGTGCTTCTTACAACGACCTTCAGCATCTGGTACAACTTCGTAACTCCCGAGTACATACCATCCACACCTGCCCGGGCGGGCCTGTGGGCTGTTTACACAGTACTACAGGGCTTGTTTGGTACCGGACTCTGGGTCATTGCGCATGAGTGTGGTCACGGCGCTTTCTCTGACTCGCGAATCATCAACGATATCACCGGATGGGTTCTGCACAGTTCGCTGCTGGTGCCTTACTTCTCGTGGCAGATTTCCCATCGAAAACACCACAAGGCCACGGGCAACATGGAGCGAGACATGGTCTTTGTGCCTAGAACACGGGAGCAGCAGGCCACTCGGCTGGGCAAGATGACCCACGAGCTAGCTCATCTCACTGAGGAGACGCCGGCCTTCACCCTCCTTATGCTCGTGCTGCAACAATTAGTTGGGTGGCCCAACTATCTCATCACAAATGTGACTGGCCATAACTACCACGAACGTCAGAGAGAGGGTAGGGGAAAAGGTAAGCACAACGGTCTGGGTGGAGGGGTCAACCACTTTGACCCCAGGAGCCCTCTGTACGAGAACTCCGATGCCAAGCTGATTGTGCTCTCGGACATTGGAATTGGCCTCATGGCTACTGCCCTTTACTTTTTGGTGCAGAAGTTTGGATTCTATAATATGGCAATTTGGTACTTTGTCCCCTACCTGTGGGTGAATCACTGGCTGGTGGCTATTACTTTTCTGCAGCACACCGACCCAACTCTCCCTCACTACACCAACGACGAGTGGAACTTTGTCCGAGGAGCAGCCGCTACCATTGACCGAGAGATGGGATTCATCGGCCGACATCTACTCCATGGTATCATTGAAACCCACGTTTTGCATCATTATGTGTCGAGCATCCCCTTCTACAACGCAGATGAGGCAACAGAGGCCATCAAACCCATCATGGGCAAGCATTACAGAGCCGACGTTCAAGACGGACCGCGGGGCTTCATTCGAGCCATGTACCGATCCGCTCGAATGTGCCAGTGGGTGGAACCTTCGGCCGGGGCTGAAGGTGCGGGAAAGGGCGTCCTGTTCTTCCGTAACCGAAACAATGTCGGCACTCCTCCAGCGGTTATCAAGCCTGTTGCTTGA |
| IgΔ9E | ATGGCCACTGAAGCGACTGCATCCATCTGGGCCGCTGTTTCCGACCCCGAAATTCTCATTGGCACCTTTTCGTATTTGCTTCTCAAGCCCATACTACGGTCATCTGGTCTGGTAGACGAGAAGAAGGGCGCCTACCGAACCAGCATGATTTGGTACAACGTCATTCTGGCCTTGTTTTCTGCTACATCTTTTTACGTCACGGCTACAGCACTGGGATGGGATTACGGATCTGGAGAGTGGCTGCGACGTCTCACCGGAGACACCCCTCAGCCCCTTTTCCAGTGTCCTAGCCGCGTCTGGGACTCGAAGCTGTTTGTGTGGACCGCCAAAGCGTTTTACTACTCCAAGTACGTGGAGTACCTGGATACTGCCTGGCTCGTTTTGAAGGGTAAGAACGTGTCCTTCCTCCAAGCCTTCCACCATTTTGGTGCTCCCTGGGATGTCTACCTCGGTATCAGACTGCAGAACGAGGGCGTGTGGATCTTCATGTTTTTCAACTCGTTCATCCACACGATCATGTACACCTATTATGGCCTTACTGCTGCTGGCTACAAGATCAAGGCCAAGCCACTCATCACAGCCATGCAGATTTCTCAGTTCATGGGTGGATTCATTCTTGTTTGGGACTACATCAACATTCCGTGTTTCCGAAGTGATAATGGCAAGGTGTTCTCCTGGGTCTTCAACTACGCCTATGTGGGCTTTGTTTTTCTGCTGTTCTGCCATTTCTTCTACAAGGACAATCTGGCATCGAAAAAGCCTGCTAAAGGGGGAAAGGCTCTTTGA |
| CcΔ8D | ATGGGTGTGAAGAAGGCTGACGAGGGCTACACTACCGTTGATCAGGGAGTTTACCCCGTCCCTGACATCCCCATCAAGGACCTGCTGGCTGCCATCCCCCCCCACTGCTTCAAGCGATCCGCTTGGCGAGGCGCCACCTACGTGTTCTGGGACCTGTTCGTGATCGGCTGCGCTTACAAGGCTGCCTCCTACCTCGACACCCTGATCAACCCCGCTACCCTTGAGCTGCCCCACCCTTACGCCTACACCGCCCTGCGAGTGGTTGTTTGGGCTCTCTATACTTTCATCGCTGGACTCTTCGCTACCGGCATTTGGGTTATTGGACACGAGTGCGGCCACCAGGCCTTCTCCGAGTCCAAGGCTGTCAACAACGCTTGGGGATGGGTCCTGCACTCCGCTGTCGGTGTCCCCTACTACTCGTGGCGAATTACCCACGCTAAGCATCATGCTTCTACCGGCCACATGACTCAGGACCAGGTCTTTGTCCCCCACACCCGATCCGAGCTGGGCCTGCCCCCCCTCAACCAGGAGGGTGAGAACCTGCTCGGATCTCGAGTGTCCGAGGTGATCAAGCGAGAGCTGTGGGACGCCCTGGGCGACTCCCCCATCGGAGCCATCATTGGATCGGCCAAGTACCTGCTTGGCGGTTGGCCCGCTTACATCCTGATGAACGCTTCTGGCCAGAAGCGATACCCCAAGTGGGTCACCAACCACTTCAACCCCAACGCTCCCATGTTCGCCCCCCACCATTACAACCAGGTCCTGCTCTCTAACCTGGGTATCATGATCTGGCTGGCTGGAATCATTGCATCCATTAACCACTGGGGCTTTTCTACCGTTCTCGTTGTGTACCTGATTCCCTACCTCTGGGTCAACCACTGGCTGGTGCTCATTACCTTCCTCCAGCATACCGACCCCCTGCTGCCCCACTACCGAGCTCCCGAGTTCACCTTCGCTCGAGGCGCCCTGGCCACCTTCGACCGATCTCTGCTCGGCGACCTGGGCCCTGTGATGGCTTGGCTGGGAGCTCACGCTACCAACGGTATTTCTGAGACTCACGTGCTGCACCACGTCTGCTCTAAGATTCCTCACTACCACGCCTGGGAGGCCTCTGAGGCCCTTAAGAAGGTTGTTAACCGAGCCGGCATCCCCACCGAGGGACGACCCGGAGGATGGCGAGAGGTCTACCGAGTGTACAAGGAGTGTAAGTTCGTCGAGGACGAGGGTGACGTCGTGTTCTTCAAGAACGCTCACGGTCTGGCTCAGATGCGACCTCAGATGCCCGAGACTTCTGCCTCCGACTCTGGCATCGAGGTGGAGAAGTAG |
| MaΔ5D | ATGGGCACCGACCAGGGAAAGACCTTTACCTGGCAGGAGCTGGCCGCTCATAACACCGAGGACTCTCTGCTCCTGGCCATCCGAGGAAACGTGTACGACGTCACCAAGTTCCTGTCCCGACACCCTGGTGGAACCGACACCCTGCTGCTGGGTGCCGGTCGAGACGTGACCCCCGTCTTTGAGATGTACCACGAGTTCGGAGCTGCCGAGGCCATCATGAAGAAGTACTACGTGGGCACCCTGGTGTCAAACGAGCTCCCCATCTTCCCCGAGCCTACCGTCTTCCACAAGACCATCAAGGGCCGAGTCGAGGCCTACTTTAAGGACCGAAACATGGACTCCAAGAACCGACCCGAGATCTGGGGCCGATACGCCCTGATTTTCGGCTCCCTCATCGCCTCCTACTACGCTCAGCTGTTCGTCCCCTTCGTCGTCGAGCGAACCTGGCTGCAGGTCGTCTTCGCCAACATCATGGGTTTCGCCTGCGCTCAGGTCGGCCTGAACCCTCTCCACGACGCTTCTCACTTCTCTGTTACCCACAACCCCACCGTCTGGAAGATCCTGGGCGCCACCCACGACTTCTTCAACGGAGCTAGCTACCTCGTCTGGATGTACCAGCACATGCTGGGCCACCACCCTTACACCAACATCGCCGGCGCCGACCCTGACGTCTCTACTTCTGAGCCCGATGTCCGACGAATTAAGCCCAACCAGAAGTGGTTCGTGAACCACATCAACCAACACATGTTCGTTCCTTTCCTCTACGGCCTTCTTGCCTTCAAGGTTCGAATTCAGGACATTAACATTCTGTACTTCGTCAAGACCAACGACGCCATCCGAGTGAACCCTATTTCTACCTGGCACACCGTGATGTTCTGGGGCGGCAAGGCCTTCTTCGTTTGGTACCGACTCATCGTGCCCATGCAGTACCTGCCCCTCTCCAAGGTCCTGCTCCTGTTCACCGTCGCCGACATGGTGTCCTCCTACTGGCTCGCCCTCACCTTCCAGGCCAACCACGTGGTGGAGGAGGTCCAGTGGCCCCTGCCCGACGAGAACGGCATCATCCAGAAGGACTGGGCCGCCATGCAGGTGGAGACTACCCAGGACTACGCTCACGACTCCCACCTGTGGACCTCCATCACCGGATCCCTGAACTACCAGGCCGTGCACCACCTCTTCCCCAACGTGTCCCAGCACCACTACCCCGACATCCTTGCTATCATCAAGGACACCTGCTCTGAGTACAAGGTTCCCTACCTGGTCAAGGACACCTTCTGGCAGGCCTTTGCCTCCCACCTCGAGCACCTGCGAGTGCTGGGCCTCCGACCTAAGGAGGAGTAA |
| PaΔ17D | ATGGCTTCCTCTACCGTTGCCGCTCCCTACGAGTTCCCTACTCTCACCGAGATCAAGCGATCCCTGCCTGCCCACTGCTTCGAAGCCTCTGTTCCCTGGTCCCTCTACTATACCGTGCGAGCTCTGGGCATTGCCGGTTCCCTTGCTCTCGGACTGTACTATGCTCGAGCCCTTGCTATCGTGCAGGAGTTTGCACTGCTCGATGCCGTCCTTTGCACTGGCTACATTCTGCTCCAGGGTATCGTGTTCTGGGGATTCTTTACCATCGGTCACGACTGTGGACATGGTGCCTTCTCGCGATCCCACCTGCTCAACTTCTCTGTTGGCACACTCATTCACTCCATCATTCTGACTCCCTACGAGTCGTGGAAGATCAGCCATCGACACCATCACAAGAACACCGGCAACATCGACAAGGATGAGATCTTCTACCCTCAGCGAGAAGCCGACTCTCATCCCCTGTCCCGACACATGGTCATCTCCCTTGGTTCGGCTTGGTTTGCCTACCTCGTTGCTGGATTTCCTCCCCGAAAGGTCAACCACTTCAATCCCTGGGAGCCTCTCTACCTGCGAAGAATGTCTGCCGTCATCATTTCCCTCGGCTCTCTCGTGGCCTTTGCTGGTCTGTACGCCTACCTTACCTACGTCTACGGCCTCAAGACCATGGCTCTGTATTACTTCGCACCTCTCTTTGGATTCGCCACCATGCTGGTTGTCACTACCTTCCTCCATCACAACGACGAGGAAACTCCCTGGTACGCCGATTCGGAGTGGACCTATGTCAAGGGCAACTTGTCCTCTGTGGACCGAAGCTACGGAGCCCTCATCGACAACCTGTCCCACAACATTGGTACACATCAGATCCACCATCTGTTTCCCATCATTCCTCACTACAAGCTCAACGAGGCCACTGCTGCCTTCGCTCAGGCCTTTCCCGAACTGGTGCGAAAGTCGGCTTCTCCCATCATTCCCACCTTCATCCGAATTGGTCTTATGTACGCCAAGTACGGCGTGGTCGACAAGGATGCCAAGATGTTTACCCTCAAGGAGGCCAAGGCTGCCAAGACCAAAGCCAACTAA |
| PaΔ5E | ATGGCTGCTCGAGTAGAGAAGCAGCAGGCACCGGCCAAGGCTGCGAAAAAGGTGGGCAGTCGTGTGGATCGGTCAGATGGATTCTTCCGAACATTCAACCTGTGCGCCCTCTACGGCTCGGCCTTCGCTTATGCATACAACAACGGCCCCGTGGACAACGACGGAAAGGGCCTCTACTTCTCTAAATCTCCTTTTTATGCGTTTCTGGTTTCTGACGCCATGACCTTTGGTGCTCCCCTTATGTATGTCATCGCCGTCATGGCGCTATCGCGATACATGGCTGACAAGCAGCCCCTCACCGGCTTCATCAAGTCCTACATACAACCTGTCTACAATATTGTCCAGATTGTTGTATGCTCATGGATGGCTTGGGGCCTGCTGCCCCAGGTTGATATTTTCAATTTGAACCCCTTCGGATTAAATAAGCAAAGAGATGCCAACATTGAGTTCTTTGTGATGGTCCATCTGCTAACCAAGTTCCTTGACTGGACCGACACTTTCATCATGATCTTCAAGAAGAACTACGCCCAGGTGTCTTTTTTGCAGGTGTTTCACCACGCAACCATTGGAATGGTGTGGTCTTTTTTGCTCCAACGCGGCTGGGGTAGCGGAACCGCCGCTTACGGTGCCTTTATCAACTCCGTTACTCATGTGATTATGTACACGCACTACTTTGTGACTTCGCTTAACATTAACAATCCTTTCAAACGGTACATTACGGGTTTCCAGCTGAGCCAGTTCGCCAGTTGTATCGTCCATGCTTTGCTAGTTCTGGCCTTTGAAGAGGTGTACCCTCTCGAGTACGCGTACCTGCAGATCTCCTACCACATCATCATGCTCTATCTGTTTGGAAGACGAATGAACTGGTCCCCACTCTGGTGTACAGGGGAAGTTGACGGTCTGGACGTCAACGTCGAGACTTCCAAGAAGGCCCAGTGA |
| IsΔ4D | ATGTGTAACGCCGCCGTGGAGAAGAAGCAGCCTCCTCGACCCTCTTGGACTAAGATCCACGGACGAGTCGTGGATGTGGCCAACTTCCGACATCCCGGTGGCAACATCCTGGACCTGTTCCTGGGAATGGACGCCACCTCTGCCTTCGAGCAATTCCACGGCCACCACAAGGGCGCCTGGAAGATGCTGCACTCCCTGCCCGAGAAGGTCGTCGACCAGGCCGACATCCCCACCCAGAACGACGAGCACGTGGCCGAGATGACCCGACTGATGACCTCTTGGCGAGAGCGAGGCCTGTTCAAGCCCCGACCCGTCGCCTCTGCCGTCTACGGCATTTGTGTGGTGCTCGCCATCATTGCTTCTGTCGCCTGTGCTCCTTACGCCCCTGTCATCGCTGGCATTGCCGTTGGCACCTGTTGGGCCCAGTGCGGCTTCCTGCAGCACATGGGAGGTCACCGAGAGTGGGGACGAAAGTGGTCCTTCGCCTTTCAGCACTTCTTCGAGGGACTTCTCAAGGGTGGCTCTGCCTCTTGGTGGCGAAACCGACACAACAAGCACCACGCCAAGACCAACGTTATCGGAGAGGACGGTGACCTGCGAACCACCCCCTTCTTTGCTTGGGACCCCACCCTCGCCAAGAAGGTCCCCGACTGGTCCCTGCGAACCCAGGCTTTTACTTTCCTCCCCGCCCTGGGTGCCTACGTGTTCATCTTCGCTTTTACTGTCCGAAAGTACTCTGTGGTGAAGCGACTGTGGCACGAGGTCGCTCTCATGCTGGCTCACTACGCCATCTTTGCCTGGGGCCTGCACGTTGCCGGCGCTACTCTGAAGGCCGGACTGACCTTCTACTGCACCGGATACGCCTGGCAGGGTATCTACCTGGGCTTCTTCTTTGGTCTGTCTCACTTCGCTGTGGAGCGAGTTCCCTCGACCGCCACCTGGCTGGAGTCTACTATGATCGGCACCATCGACTGGTCCGGTTCTTCCGCCTTCTGCGGCTACCTCTCTGGCTTCCTCAACATCCAGATCGAGCACCACATGGCTCCTCAGATGCCCATGGAGAACCTGCGACAGATCCGAGCCGACTGCAAGGCTAGTGCCGAGAAGTTTGGACTGCCCTACCGAGAGATGTCCTTCCTCGCTGCCGTTAAGCTCATGATTAACGGCCTCTACCAGACCGGTAAGGAGGAGCTGAAGCTGCGATCTGACCGACGGAAGTACTCCCGAGCCCAGGCCTACCTGGGAGCCGCCAGCGCCGTCGTTGAGACTCTGAAGGCTGATTAA |
| ScAgo1 | ATGTCCTCTAACTCTGAAGAGAACTCTCAGGTGCCTCCTCTGGACGCCACCGCCGCTGCCACCAAGCCTAAGAAGGCCAAGAAGCCCAAGGTTAAGAAGCCTAAGGACTCTGCCGAGGCCTCTTCTTCGCCCGCTGCCGAGGGCACCGCCGAGGCTAAGCCCAAGAAGGCTAAGAAGTCTAAGACCAAGAAGTCGAAGGAATCCGCCGAGGTGTCTCCCGCTCCTGCCGACGAGACTACCTCTGCCGGCGTGGACGCAAAGCCAAAGAAGGCAAAGAAGAGTAAGGTCAAGAAGCCAAAGGATTCCACCGAGTCCTCGCCTGCTCCTTCTAACGAGCCTCCTGCCGCTGAGGTGGCCGCCGAGGACGCCAAGTCCAAGAAGGTGAAGAAGCCGAAGGCAAAGAAGCCGAAGGAATCTACTGAGTCGAGCCCCGCTCCAGGCCAAGAGGCTGCCGCCACCGAGGGCGCTACCGAGGATAAGCCTAAGAAGGTGAAGAAGAGTAAGGCTAAGAAGGCCAAGGAATCTGTCGAGTCGTCCCCTGCTGCTACCGAGTCTGTGTCTGAAAAGACCGCAAAGAAGTCGAAGAAGCCCAAGGCCAAGAAGTCTACCTCTCCTGAGACTACCGAGGAAATCACCGAAGAGTCTACCGAATCTAAGGAAAAGAAGACCAAGACAAAGAAGCCCAAGGAAAAGAAGAGTTCTCCCTCTACCGCCACCTCCACCGCTGCCTCTAAGCCCGTGACCTCTATCGCCGGCGTGACCATTCCTGGCAAGACCTTCGACCTGACCGACGTGCCTCCTTCGAAGCCCGCTCCTAAGATGGTGCCCGAGGCCTACAAGCTGCAGACCCGAGTGGACTACGGCACCAAGGGCACTAAGGTGGACGTGCTGACCAACCACATCCTGCTGTCTGTGGGCGACGACGTGCCCCAGGACGAGCGAGCCTCTCAGCTGGACCCCTGGTGGAAGTCTGCCTTCGTGTACACCTACAACATCACCTTCGCTGTGCCCCAGTCTAACTCTCCCCGAAAGGGCCCTGCTCCTGCTCTGTCGAAGCCGAAGAAGTACGAGCTGGTCGAGTCTCTGTTCCACGAGGACGAGACTCTGTTCAAGTACAAGGACCGAATCTCTTTCAACGGCGAGGACACCCTGTACTCGCACGTGCCCCTGGAAGAGTTTACCCTGTTCGACGGCTGCTGGGACGTGTCTAACAAGCAGAAGAAGCGACGACAAGAGGTCGTGGGCCTGAACTCTCGAGCCAAGGAAATCAACGACCTGGCCGCTCAGGTGACCCTGAAGTTCGCCGACAAGGTGCCTCTGGGCGACATCTACAAGGCCACCACCTCTAAGGACCCCGAGGAACAAGAGAACAAGATGGCCAACGCTGACAAGGTGGCCCTGCTGTCCCTGATGGGCGTGAAGTTCCTGAACACCAAGGAACAGATCTTCCAGCTGAACGGCAACAAGTTCTTCATCTTCAACGAGCACGCCATTGCTACCCCTTTCCAGATCGGCGGCTTCCTGATGCACGGCTTCACCGTGTCTCTGCGATACGCCTACGGCTCTGTGCTGCTGAACACCGTGAACGTGTGTCTGCCCTTCGTCAAGTGGACCAAGTACCTGCCTGGCGACGCTAAGTTCAAGGAAAACGAAAAGACCCAGTACTCTCTGCTGGACTGGATCATCGAGTGTATGCACCAGGCCTCTGCTCAGCGAGGCCAGAAGCTGCGAGGACCTCCTTCGGCCAAGGACATTAACTTTTTCATCGACAAGAACCGAGACATCAAGGACCTGCTGAAGGGCCTGAAGTGCTACCGACCTTACATCAACTACTCTGTGAACCCCGACGGCACCCCTAAGCCTCCAAAGAAGATGCAGGCCAAGGGCATCGTGGGCTTCGTGCGAGAGACTCCCGACTCTATGAAGTTTCGAACCCTGCCTTCTAACATGGAAAAGAACGGCGTGCCCAAGCCTGGCGAGAAGGAAATTATGGTCACCACCACCGCCTACTTCGCCAAGAAGTATGACATCAAGCTGAAGTACCCCGACGTGAAGATGGTGTCTCTCGGCGGCTCTAACGTGGTGCCCGCCGAGTGCCTGACCATCGTGCCCGGCCAAAAGCTGAAGGGACTCGTGTACGACGAGAAGGCCGTGATCGACTTCACCGCTCTGCGACCCTCTGAGAAGTTCCGAGCCATCACCAACCTGGCTCTGCCCGCCATCAAGCGAGCCCTGTCCACCGAGGAAGAGAACGCCAAGGCTCCCCACGACTCTGGCTACACCTTTATGAAGGTGCCCTCTCGAGTGATCGACGCTCCCGTGGTGCAGTTCAAGAACACCACCGTGACCTACGTGGACAAGCCCTTTGGCACCAAGAACGGCAAGAACAACCACGAGGAAACTAAGGGCAACTGGAACCTGAAGGACCACAAGTTCATCACCGTGCCTAAGGAACCCATGCATCTGCGAGCCATCTTCATCAACGACTCCGACAAGTCTCCTCCTGTGTCCGTGATGGACGAGCTGAAGGCCTCTCTGTCTAAGTTCGCTGAGGACGTGGCCGACGTGGGAGTGAACTTCGACGTGTCCATGGCTCCCATCCTGATCAACAACTTCAACGCTCCCATCAAGAAGGTCACCGGCGGTTTCGGAGGCCGAGGCGGCCGAGGTGGAAGAGGCGGAAGAGGTGGTCGAGGCGGCAGAGGCGGCCGAGGCGGATTCGGCGGAGGACGAGGCGAAACCACCTACGAGCTGACTCCCGGCGAGGAAAAGCTGCGACATCTGCTGGCCAACGTGCCCGAAAAGACTTACGTGCTGTTCGTGCTCGGACGAGGTGACGACTCTGCCATCTACAACCGACTCAAGTACCTCGCTGACCTGACCTACGGCGTGATTAACAACTGCGTGATCTGGAACAAGTTCCGAAAGTGCTCTACCCAGTACAACGTGAACGTCGTCATGAAGATGAACCTCAAGCTCGAGGGCGCCAACCACTCTCTGTGTGCCGAGGACATCAACCTGCTCAAGGACGAGAAGTCTGGACTGCCCTTCATGATCCTGGGAGCCGACGTGACTCACTACCCCGAGAAGGACCAGAACTCTATCTCTGCCCTGGTGGGCTCTTTTGACGACAAGTTCGCTCAGTTCCCCGGCTCTTACATGCTGCAGTCTGGACCCGGCGAAGAGATCATTGCCGGCATCGGCAACATGGTGCTGCAGCGACTGAAGCTGTACCAGAAGCACAACAACGGCAAGCTGCCACCTAAGATCCTGTTCTACCGAGATGGCGTGTCTGAGTCTCAGTTCTCTCAGATCGTGCAGATCGAGGTCAAGGGACTGAAGCAGGCCCTGAAGAAGTTCGGCTCTGAGCTGAACAAGGGCGTGAACTACAACCCCTCTGTGACCACCATCTGCGTGGTGAAGCGAAACCAGATCCGATTCATGCCCCTCGAGCAGAACGCCATTAACGAGAAGGGCGAAGTCGCCGCCGTGCAGTCTTTCGAGAACGTGATGCCCGGCACCGTGGTGGACCGAGGAATTACCTCTTCTGCCCACTTCGATTTCTTTCTGCAATCTCAACAGCCCCTGAAGGGCACCGGTGTGCCCTGCCACTACTGGTGCATCTACGATGAGAACCAGTTCAACTCTGACTACCTGCAGCAGGTCACCCACGCTCTGTGTTACCTGTTCGGCCGATCTTCTACCTCTATCAAGGTGGCTTCTCCCGTGTACTACGCCGACCTGCTGTGCGAGCGAGGCGCCGCTTTCTTCAAGGCCAACTTCGAGCTGGCCCAGTACGAGTTCTCTAAGGAACGAAAGAACCGGGACGACGTGATCCCCACCGGAAAGCTGCTGCAGCCCGTGCACAAGAACGTGACCGACATCATGTACTACATCTAG |
| ScDcr1 | ATGAACCGAGAGAAGTCTGCCGACCTGTCTAAGACCTCTGACGCTCCCTACAACGAGCTGGACGCCAAGAACCTGCGAAACTTCTACAAGGTGCAGAACGCCTGCGCTCAGCTGCGAGAGTCTATCAAGGTGATCTACGAGAACGGCCTGTCCTCTGACCAGCTGAACACCATGGCCAAGCACGGCAACGACCTCGAGAAGTCTATCGCCAACTCTCCCGCCATGTCTGTGGCCTCTTGCCTGAACCAGGTGCGACCCACTCTGGACATCAAGAACATCTTCGACCACTACAAGTTCGAGAACAACTCTTCTCCTGTGGACCCCTACGTGCACTACCCCGTGTGTTCTGACCAGAACCTGGAAAACCTGGCCTTCATCCACCGATCTCTGCCCAACATGAACGTGAAGCTGACCGAGCTGCAAAAGACCGTGATGTCTAACGAGCGACTCGAGTTCCTGGGCGACTCTTGGCTGGGCGCCCTGGTGGCCTACATCATCTACAAGAAGTACCCCTACGCCAACGAGGGCGCTCTGTCCAAGATGAAGGAAGCCATCGTTAACAACAACAACCTTGAGAAGATCTGCGAGAAGCTGGGCTTCAAGGAACGACTGAAGGAAAACATTCCCCGATCCTCTATGAAGATTAAGGACCGACTGACCAAGAACTACGCCGACTGCGTCGAGGCCTACATTGGAGCCCTGGTGATCGACCGATTCTCTACCGAGTTCAACGACGTGGCCCTGTGGCTCGAGGAACTGTCTGAGGAACACTTCATCGAGCTGGGCCCCATGATGGTGAAGGAACCCCTGAACAAGAACGCCAAGGGCGAGCTGGGAGCCTTCCTGCAGTTCAACAACATCGGCGCCAAGATCTCTTACAAGCGACTGAACGACAAGTCGCCCTTCAAGGTCGAGGTGCGACTGGGCAACAACCTGCTCGGCATCGGCGACGGCTCTAACGTGCGAGAGGCCGAGCAGCGAGCCGCCATGGAAGCCCTGGCTCAGCCCAAGCTGATCCAGAAGTACTCTCTGCACGACATCGAGCTTGAGCGAGGCATGATCGAAGAGGCCGACAACGTGCCCCAGCTGCTGAAGTCCGCCGAGATTCCCAACCAGACTCCTCAGTCTCACCACTCTATCACCGAGGAAATCGACGAGGAAGAGGGCCTGACCTTTCCTCACTCTAACCCATTTCTGAAGGGCACCCCTCCTGCCGTGCTGCCCGAGATCGTGACTAACCAGATTCCACAGGCCTCCGCCGACCACCAAGAGCAGAACGTGCCCGACACCGACAAGATCGTGAACGACGTGATGGAACGAATGTCTAAGATCCTGTCTGTGATGGTGTCTGAGGCCGTGTCTAACGCCCTGGGACGACCCGCTCCTAAGAACGCTCCCATTCCTATTCCAGTGACTACTCCCGTGTCTCCCCTGGCCACCTCTCCTGTGTCCGTGTCTGTGCCCGCTCCTGCTCCTGCTGTGGCCGTGACTCCCTCTGTGGTGAACAACCCTCCTCAGCCTAAGATGGTGACCCAGCCTGTGTCTACCAACCTCAACAAGACCCCTGAGGTGGCCCTGCTGAACTCTGTGTACGACAAGGAAGCCTCTGGCCGACTGTACGCCCTGCTGGGCAAGTACAAGCTGTACCCCGAGTACAACACCGAGCAGCTCGGCATGACCGACTTCTACACCGTGTGCTTCATCAAGGGCGTGGGCGTCGAGATCGGCAAGGGCCACGGCCGATCTAAGAAGATCTCTCAGCACAAGTCTGCTGAGGACGCCCTGAACGGCAAGGCCCTGAAGGAATACCTGCGACACTGTAACAACCTGTAA |

**Supplementary Table 5. Primers used for overexpression plasmid construction in this study**

| **Primer** | **Sequence (5'→3')** |
| --- | --- |
| P2902-eGFP-F | AGCCGAGCGCGGGAGGGAAGATGGTGAGCAAGGGCGAGGA |
| P2902-eGFP-R | TGACATAACTAATTACATGATTACTTGTACAGCTCGTCCATGCCG |
| P2902-RdRP-F | AGCCGAGCGCGGGAGGGAAGATGGCCGTCTCGCTCAACGA |
| P2902-RdRP-R | TGACATAACTAATTACATGACTAAAAGTTGTTGGCCGTGAG |
| P3626-SsΔ9D-F | TCAAATCAGCCGCAAGGAAAATGACCGAGCAATTGGACAC |
| P3626-SsΔ9D-R | TGACATAACTAATTACATGACTAGTTAAGCTTCTCCTTGTCCAAG |
| P2902-GGPPS-F | AGCCGAGCGCGGGAGGGAAGATGGCCTTTTCCACGAGACCG |
| P2902-GGPPS-R | TGACATAACTAATTACATGATCAAATATCGCAGTCGTCAACTTCTTC |
| P3626-PSY-F | TCAAATCAGCCGCAAGGAAAATGGCGCGCATGGGCGTCAGC |
| P3626-PSY-R | TGACATAACTAATTACATGACTAGAAGGTCTTTAGGAAGTTATTCTTGAAG |
| P2520-LCYB-F | CAGCAGCAGCAGCAGGAACAATGGCGCGCAGGGCGTCGCG |
| P2520-LCYB-R | TGACATAACTAATTACATGATCAAGCATTCTTGTACAGCG |
| Int1-UP-F | TATCCTGTCAAACAGGATCCCGCCCGCAGACGCCGCCTTC |
| Int1-UP-R | CCTTCAGTTTAAACTATCAGCCACATCATGAGCGTTCGCG |
| Int1-DN-F | GCAGACACTCCCGGAGACAATCAAGTAGGTTCAAGCTTTC |
| Int1-DN-R | AATTGAATTTTTCTCGACACTAACTTGACTTGCTGCGCTGC |
| Int2-UP-F | TATCCTGTCAAACAGGATCCCTACCTAGGAGGCAATAGGCCC |
| Int2-UP-R | CCTTCAGTTTAAACTATCAGAGCGCCGTAGCGCCCTCGTG |
| Int2-DN-F | GCAGACACTCCCGGAGACAAAACAAACGCACTCGCGAACA |
| Int2-DN-R | AATTGAATTTTTCTCGACACATCAAGACTGCTCTCGTCATAACA |
| Int3-UP-F | TATCCTGTCAAACAGGATCCCGGGGACGGCCTCGTCGTCG |
| Int3-UP-R | CCTTCAGTTTAAACTATCAGGTAAACTCATCACTGCCATGGAGATTAAATAACGTCTAGGCCT |
| Int3-DN-F | GCAGACACTCCCGGAGACAACCCCTCCAATCCAAAGCTATCTCAT |
| Int3-DN-R | AATTGAATTTTTCTCGACACAGCCAGGGAATGTCGTACCC |
| Int4-UP-F | TATCCTGTCAAACAGGATCCGGGCGCGACCACTCCAAGAG |
| Int4-UP-R | CCTTCAGTTTAAACTATCAGACACGCGTTGCAAATATCAATAAGCG |
| Int4-DN-F | GCAGACACTCCCGGAGACAAGCTGATTCCTGCGAGAGACC |
| Int4-DN-R | AATTGAATTTTTCTCGACACTGCTGACCTTCCTATGCTCGTA |
| InA-1-UP-F | TATCCTGTCAAACAGGATCCAAGTAACCTCCTAATAATCTGACC |
| InA-1-UP-R | CCTTCAGTTTAAACTATCAGTTCGGGTGTAAGTAATGACAC |
| InA-1-DN-F | GCAGACACTCCCGGAGACAACCAATGAGCTACATTGTGCCATGAC |
| InA-1-DN-R | AATTGAATTTTTCTCGACACGCGCATGGGATACCCCGTCTGGAA |
| XPR2-UP-F | GTAAAACGACGGCCAGTGAATTCCTTCGGTCTGTCAGTATCAC |
| XPR2-UP-R | CATGCACCACTGGAAGATCCGGGAATTCGAATTCGGCAGTCGCATG |
| XPR2-DN-F | CGCAAGTGAGATGCCCGTGTCGGGCCCGTCGATGGGGTTTATAT |
| XPR2-DN-R | GATTACGCCAAGCTGCGGCCGCTTGCGTGAGGGTGAGCGTCCTG |
| InTF3-UP-F | ATTACGCCAAGCTATTTAAATGGGGCGCTCCAGTGGGCCG |
| InTF3-UP-R | CTCTAATTAAGGTACCAAGCTTCTGGGGACCGAGAATTAG |
| InTF3-DN-F | ATCCGGGAATTGCGGCCGCATACGCGAGTTCCCAAAGCAG |
| InTF3-DN-R | CGGCCAGTGAATTATTTAAATGTGCTGTTCAGAAGCCGGC |
| IntC-UP-F | AAAACGACGGCCAGTGAATTCAGGTTGTCGATGATATGCTG |
| IntC-UP-R | CACTGGAAGATCCGGGAATTAGGACAACTCCTGAGACATAGT |
| IntC-DN-F | GCTTATTAAGGTACCAAGCTTCGTGGTTCATGTCCTTCATC |
| IntC-DN-R | GACCATGATTACGCCAAGCTGTCGAGTTGCGGCTCGAGAGCC |
| Sc-shRNA-TEST-1 | ATCTGATCCAAGCTCAAGCT |
| Sc-shRNA-TEST-2 | TCTAGAGTCGACCTGCAGCATG |
| yl-shRNA-TEST-1 | TACCGGGGTGTGTTCTGTGG |
| yl-shRNA-TEST-2 | GCTGTTCCAGTCAATCAGGG |
| TEST-1 | GCCTGTCGCGTAACTTAGGA |
| TEST-2 | TAACACATTGCGGACGTTGC |
| TEST-3 | GCCGCAGCTTGGCGTAATCA |
| TEST-4 | GACTTGAAGCTTGGTACCTTAATG |

**Supplementary Table 6. RT-qPCR primers used in this study**

| **Primer** | **Sequence (5'→3')** |
| --- | --- |
| ***Schizochytrium* sp.** | |
| β-actin-F | TGTCCTCACGCTCAAGTAC |
| β-actin-R | GAAGGTGTGGTGCCAGATG |
| PEX10-F | TCCGGTTGTGCGTGGAATTA |
| PEX10-R | CCTCATCAGCATCGTCGTCA |
| ACOX1-F | TGGCGCTCTCTTGTTGTCAT |
| ACOX1-R | GCGTTGCAGCATACAGGATG |
| CPT1-F | CAAGACGCTCAAGCCAGAGA |
| CPT1-R | TGTAGCTAATGCCGTAGCCG |
| LCAD-F | CAAGGTCAGCGATCCCAAGT |
| LCAD-R | GAACCATCGTCGTTGATGCG |
| PLD1-F | CAGCTGACTGAATCCATAA |
| PLD1-R | AGCTACGGGAATTCAGAC |
| TGL-F | AGCAGATTTTCCTCACGCCA |
| TGL-R | ACTTTTGCTCGCTTGCCTTG |
| DGL-F | GGAAGTGACCTCATTCCGCA |
| DGL-R | TTGTTGACTTTGCTGCGACG |
| GPAT1-F | AGATGCAAAAGCCAAAGCCG |
| GPAT1-R | GAGAAAAGGTAGGCTGCCGT |
| DGAT1-F | CTCTTCCTCGCCGAGAATCC |
| DGAT1-R | TATGGCGGTCGTAAAGGAGC |
| LPAT1-F | TCCCTTTGTGCTCTCAGCTG |
| LPAT1-R | ATGTCGCCAGAGTGATGCAA |
| FAS-F | CTACGAGTTCGCGCAAATGG |
| FAS-R | GGGACTCCACAAATCCAGCA |
| ORFA-F | AAGGTCATCATGGCGCTCAA |
| ORFA-R | TTGATGGGCGTGTTGTCGTA |
| ORFB-F | ATCCCACTCCCAGAAAAGCG |
| ORFB-R | GCTGGAAGTCGACCTCAACA |
| ORFC-F | CTGGTTCTTCTCGTGCCACT |
| ORFC-R | CAAAGGTGGGGTTGGCAATG |
| eGFP-F | AGGACGACGGCAACTACAAG |
| eGFP-R | GATGTTGCCGTCCTCCTTGA |
| GGPPS-F | CAGGCAAGTGTCCTAGCGAA |
| GGPPS-R | CGCGATTCGGAAAAGCTCTG |
| PSY-F | ATTTCAACCTCGAGCTCGCA |
| PSY-R | CTGCAAAGGCCTCTCCAAGA |
| LCYB-F | TTCAAGCTGTTTGCACTCGC |
| LCYB-R | CAGAAGGGTCTGCTCAACGT |
| ***Yarrowia lipolytica*** | |
| G6PDH-F | GTGGATACTTCAACGACAT |
| G6PDH-R | ATGGCTAGAATGGACAGA |
| ylC16E-F | GTTCACGTGACTCCCTCTGG |
| ylC16E-R | CTGGAGCATTGAGGACGGTT |
| PaΔ17D-F | CGAGAAGCCGACTCTCATCC |
| PaΔ17D-R | TCTTCGCAGGTAGAGAGGCT |
